# Scaling Catalytic Contributions of Small Self‐Cleaving Ribozymes

**DOI:** 10.1002/anie.202207590

**Published:** 2022-09-02

**Authors:** Michaela Egger, Raphael Bereiter, Stefan Mair, Ronald Micura

**Affiliations:** ^1^ Institute of Organic Chemistry and Center for Molecular Biosciences University of Innsbruck Innrain 80–82 6020 Innsbruck Austria

**Keywords:** Deazapurines, Oligoribonucleotides, RNA Catalysis, RNA Modifications, RNA Solid Phase Synthesis

## Abstract

Nucleolytic ribozymes utilize general acid‐base catalysis to perform phosphodiester cleavage. In most ribozyme classes, a conserved active site guanosine is positioned to act as general base, thereby activating the 2′‐OH group to attack the scissile phosphate (γ‐catalysis). Here, we present an atomic mutagenesis study for the pistol ribozyme class. Strikingly, “general base knockout” by replacement of the guanine N1 atom by carbon results in only 2.7‐fold decreased rate. Therefore, the common view that γ‐catalysis critically depends on the N1 moiety becomes challenged. For pistol ribozymes we found that γ‐catalysis is subordinate in overall catalysis, made up by two other catalytic factors (α and δ). Our approach allows scaling of the different catalytic contributions (α, β, γ, δ) with unprecedented precision and paves the way for a thorough mechanistic understanding of nucleolytic ribozymes with active site guanines.

## Graphical abstract

General base knockout in nucleolytic ribozymes using deazaguanosine‐modified mutants paves the way to scale the relative contributions of the four main strategies (α, β, γ, δ) applied in ribozyme general acid base catalysis.

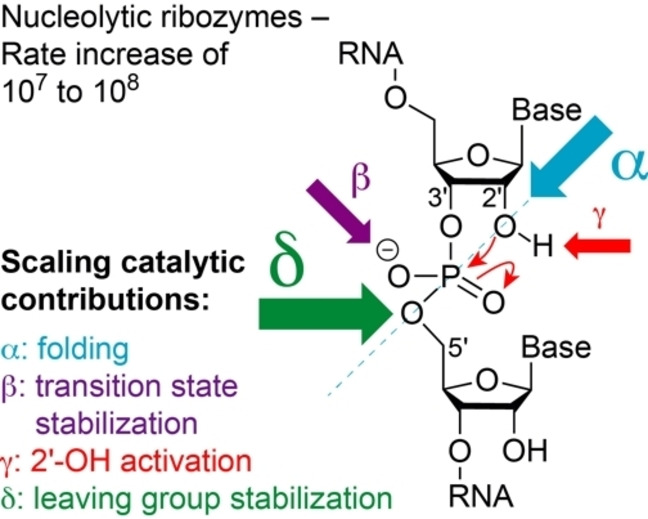

## Introduction

**Figure 1:**
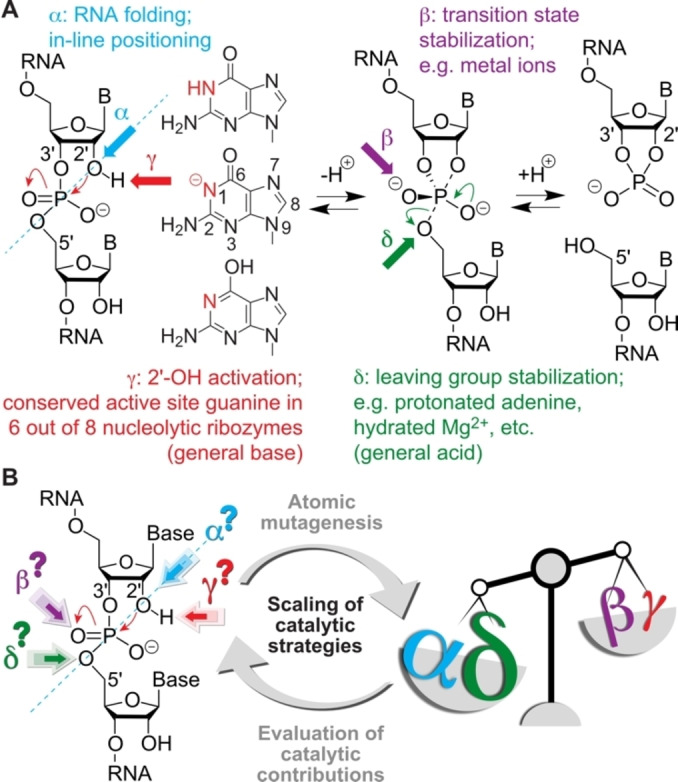
Small self‐cleaving nucleolytic ribozymes. A) Reaction scheme for RNA phosphodiester cleavage with catalytic factors α, β, γ, and δ as indicated. In 6 out of 8 ribozyme classes (hammerhead, hairpin, Varkud satellite, twister, *glmS*, pistol, hepatitis delta virus, twister sister) a guanine is attributed as general base.^[8]^ How guanine handles catalysis remains elusive; this study aims to shed light on this question involving novel 1‐deazapurine nucleosides. B) Scaling of catalytic strategies by dissecting based on atomic mutagenesis.

Small self‐cleaving ribozymes are crucial players in the life cycle of cellular RNAs, for example, they control replication of satellite and pathogenic RNAs.[^1^, ^2^, ^3^] From a mechanistic point of view, site‐specific cleavage of their phosphodiester backbone is characterized by a nucleophilic attack of the 2′‐OH on the adjacent 3′‐phosphate, leading to cleavage products carrying 2′,3′‐cyclic phosphate and 5′‐hydroxyl termini (Figure 1A).[^4^, ^5^, ^6^, ^7^] Adjacent nucleotides and metal ions facilitate the cleavage reaction through general acid‐base chemistry.[^8^, ^9^, ^10^, ^11^, ^12^] Mechanistic roles for nucleobases include proton transfer, electrostatic catalysis, and hydrogen bonding with the transition state.[^13^, ^14^, ^15^, ^16^, ^17^] The four nucleobases have p*K*_a_ values far from neutrality and so are not optimal for general acid‐base catalysis.^[18]^ The RNA environment however can give rise to shifted p*K*_a_ values[^19^, ^20^] and to “reverse protonation”, namely protonation opposite to expectations for the free catalytic residues. This concept was originally defined for protein catalysis (lysine, glutamic acid)^[21]^ but also holds true for ribozyme catalysis,[^22^, ^23^] meaning that A, C, and G are catalytic in ribozymes in charged states of A^+^, C^+^, and G^−^, respectively, although the corresponding free nucleotides are neutral in aqueous solution at pH 7.^[24]^ It has been associated to several ribozymes (e.g. hairpin ribozyme,^[25]^ Varkud satellite (VS) ribozyme,^[2]^ twister ribozyme[^26^, ^27^]) using anionic general bases and cationic general acids, respectively.

The catalytic contributions to phosphodiester cleavage have been categorized into for major strategies (Figure 1A): 1) In‐line positioning of the attacking 2′‐OH to the to‐be‐cleaved P−O bond (α‐catalysis); 2) Activation of the 2′‐OH group by a general base (γ‐catalysis); 3) Transition state stabilization (β‐catalysis); 4) Stabilization of the 5′‐O leaving group by a general acid (δ‐catalysis). It has remained an open challenge to scale these different contributions for individual ribozymes by experimental approaches (Figure 1B). Here, we demonstrate an approach that involves atomic mutagenesis for precise “general base knockout” to allow weighting of the different catalytic factors with unprecedent precision.

## Results and Discussion

**Figure 2:**
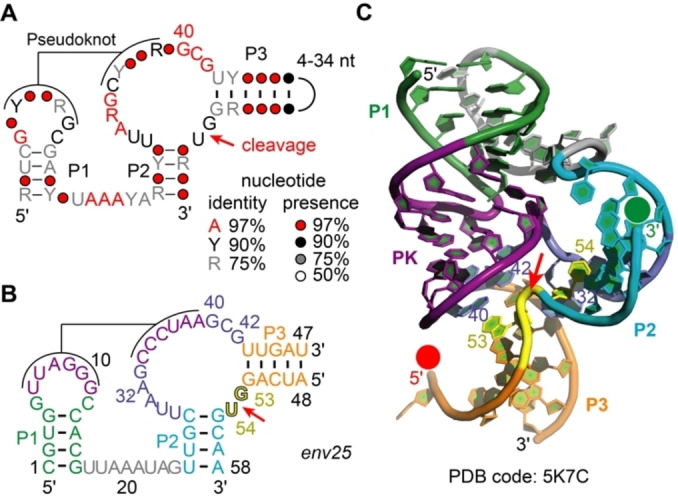
The pistol ribozyme class. A) Consensus sequence and phylogenetically derived secondary structure; Y pyrimidine, R purine. B) Representative pistol sequence (*env25*) used in the present study. C) Three‐dimensional structure of the *env25* pistol ribozyme (PDB ID 5K7C).^[21]^ Red and green spheres at 5′ and 3′ termini of the substrate strand indicate positions for fluorophore attachment; red arrow indicates site of cleavage.

Pistol ribozymes (Figure 2A,B) represent an abundant ribozyme class in diverse organisms that was discovered by comparative genomic analysis, together with three other classes, known today as twister, twister‐sister and hatchet.[^28^, ^29^] For pistol ribozymes, structure‐function studies shed some light on the nucleobase‐ and metal ion‐specific catalytic strategies of these ribozymes. In particular, X‐ray crystallographic studies of the pistol ribozyme (resulting in structures of the pre‐cleavage[^30^, ^31^, ^32^] (Figure 2C), and post‐cleavage states,^[33]^ and a transition state mimic^[33]^), as well as structure‐function analysis by targeted mutagenesis provided a framework for the identification of putative, catalytically significant structural moieties.[^29^, ^30^, ^31^, ^32^, ^33^, ^34^] Among them, a hydrated Mg^2+^ ion, innersphere‐coordinated to the N7 of G33, stands out through participation as general acid.^[34]^

In contrast, the role of the general base is less clear. Although all available crystal structures suggest an active site guanosine (G40) to embark on this role,[^30^, ^31^, ^32^, ^33^] targeted atom‐specific mutagenesis to evaluate this hypothesis lags behind. Here, we set out to explore the mechanistic role of guanine as general base in the pistol ribozyme class with novel deazaguanine mutants that lack the characteristic properties of the pyrimidine nitrogen N1 at the Watson–Crick face. Thus, by completing the set of atomic mutagenesis experiments toward the general base in context with previously reported results from mutagenesis studies, we aim at scaling the four major catalytic strategies. We exemplify this undertaking for the pistol class in detail, additionally confront our model with a recent study on the class of twister, and thus pave the way for weighting the catalytic factors of the remaining nucleolytic ribozymes with guanine as putative general base in their active sites.

### RNA Synthesis and Real‐Time Fret Cleavage Assay

To achieve our goals, synthetic hurdles had to be taken first; those concerned access to the phosphoramidites of 1‐deazaguanosine (c^1^G), 3‐deazaguanosine (c^3^G), and xanthosine (X) needed for RNA solid‐phase synthesis. Since only for c^3^G containing RNA synthetic procedures were published,[^35^, ^36^] we have developed novel protocols for c^1^G and X modified RNA.[^37^, ^38^] A further requirement for our undertaking was the set‐up of a reliable and robust real‐time assay for direct monitoring of pistol ribozyme cleavage. We decided for a fluorescence‐resonance‐energy‐transfer (FRET)‐based assay^[39]^ using a substrate strand with the donor fluorophore disulfo‐Cy3 at the 3′ end and the acceptor fluorophore disulfo‐Cy5 at the 5′ end (for details of preparation and FRET assays see the Supporting Information (Supporting Methods, Supporting Figure S1, Supporting Tables S1 and S2)).

### Ribozyme Folding and Substrate Annealing

**Figure 3:**
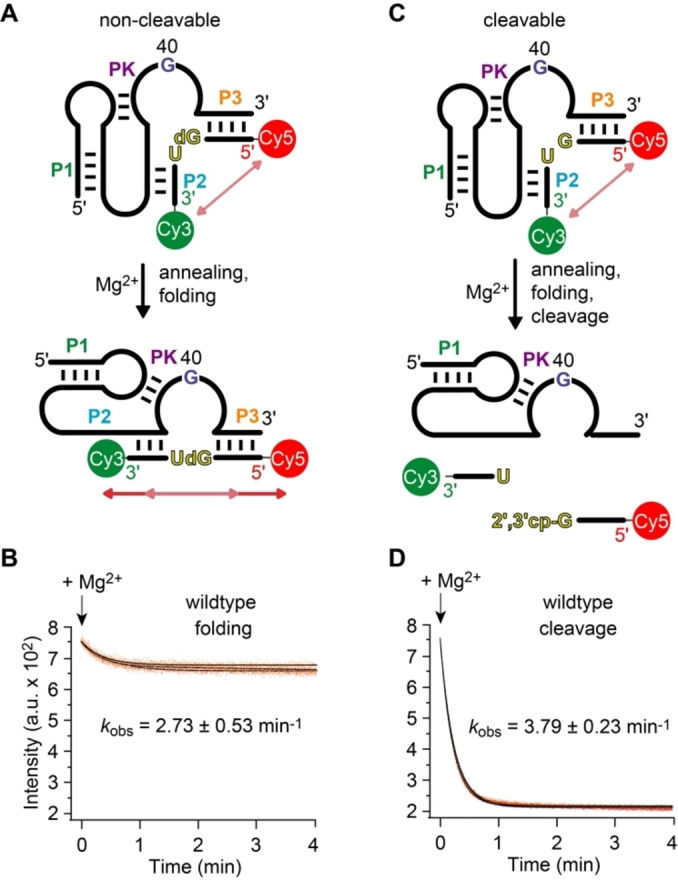
Pistol ribozyme FRET assay. A) Set up with non‐cleavable dG53 substrate to follow annealing and folding. B) Exemplary experimental FRET trace for (A). C) Set up with cleavable substrate to follow cleavage. D) Exemplary experimental FRET trace for (C). Conditions: *c*(RNA)=0.5 μM of each RNA strand (1 : 1 ratio); 10 mM MgCl_2_, 100 mM KCl, 50 mM MOPS, pH 7.5, 25 °C.

Figure 3A illustrates the concept of this assay for the uncleavable dG53 substrate strand, lacking the 2′‐OH group for attack at the scissile phosphate. Hence, upon addition of Mg^2+^ the ribozyme can fold, however, it cannot cleave because of the absence of the nucleophile. The corresponding FRET signal responded by a small decrease (Figure 3B). This reflects Mg^2+^‐induced pistol ribozyme folding by annealing the substrate strand into an extended conformation with the fluorophores slightly further apart from each other compared to the average conformation of unbound (or incompletely bound) substrate. This is consistent with the crystal structure, showing a distance of about 48 Å distance between 5′‐ and 3′‐termini of the substrate strand in stretched conformation (Figure 2C). The rate of the observed FRET decrease followed monoexponential behavior, where the estimated folding rate, *k*_obs_, was 2.73 min^−1^ at 25 °C in the presence of saturating Mg^2+^ concentrations (10 mM).

### Wildtype Ribozyme Cleavage (G40)

A decrease in FRET was also observed for the cleavable G53 pistol ribozyme upon Mg^2+^ addition (Figure 3C, D). This time, the amplitude of FRET response was significantly larger (by a factor of ≈4). This is consistent with cleavage of the substrate strand and dissociation of the cleavage products from the ribozyme. It is also in line with previously reported high cleavage yields.[^29^, ^30^, ^40^] The time course of the observed process followed monoexponential behavior, and the estimated rate of cleavage, *k*_obs_ was 3.79 min^−1^ under otherwise same conditions. This is slightly faster than the rate measured for the non‐cleavable dG53 pistol construct, and thus we infer that the 2′‐OH of the substrate likely contributes to proper annealing and folding into the active conformation.

**Figure 4:**
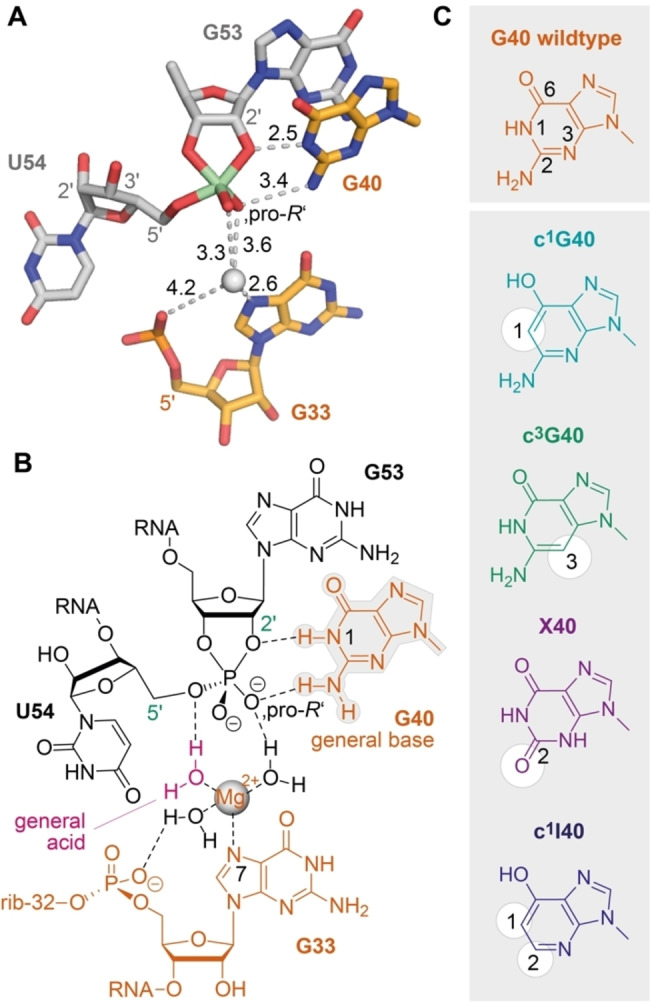
Atomic mutagenesis to shed light on the role of G40 in pistol ribozyme catalysis. A) Crystal structure of the active site vanadate transition state mimic (PDB ID 6UEY). B) Chemical structures of the active site nucleotides emphasizing the interactions of potential key players in general acid base mechanism in the transition state. C) Set of mutations used in this study to evaluate the impact of G40 in γ‐ and β‐catalysis relative to δ‐catalysis (hydrated Mg^2+^ coordinated to N7 of G33).

Having a robust real‐time cleavage assay in hands, we next focused on the major aim of our study to shed light on the impact of G40 in catalysis (Figure 4). This guanosine is phylogenetically strictly conserved in sequence[^28^, ^29^] and its role in general acid base catalysis is widely accepted because of the positioning of its nucleobase observed in the crystal structures (Figure 4A, B). The N1‐H of G40 is in perfect distance to the (modeled) attacking 2′‐O nucleophile of G53 in the precatalytic structures (PDB IDs 5K7C, 5KTJ, 6R47),[^30^, ^31^, ^32^] and importantly, also observed in direct interaction (2.5 Å distance) with the 2′‐O of the transition state mimic (PDB ID 6UEY)^[33]^ (Figure 4A). Nevertheless, how G40 activates the attacking 2′‐OH (generally referred to as γ‐catalysis[^9^, ^41^]), and to what extent has remained elusive.

### Lacking the General Base Key Functionality (c^1^G40, c^1^I40)

**Figure 5:**
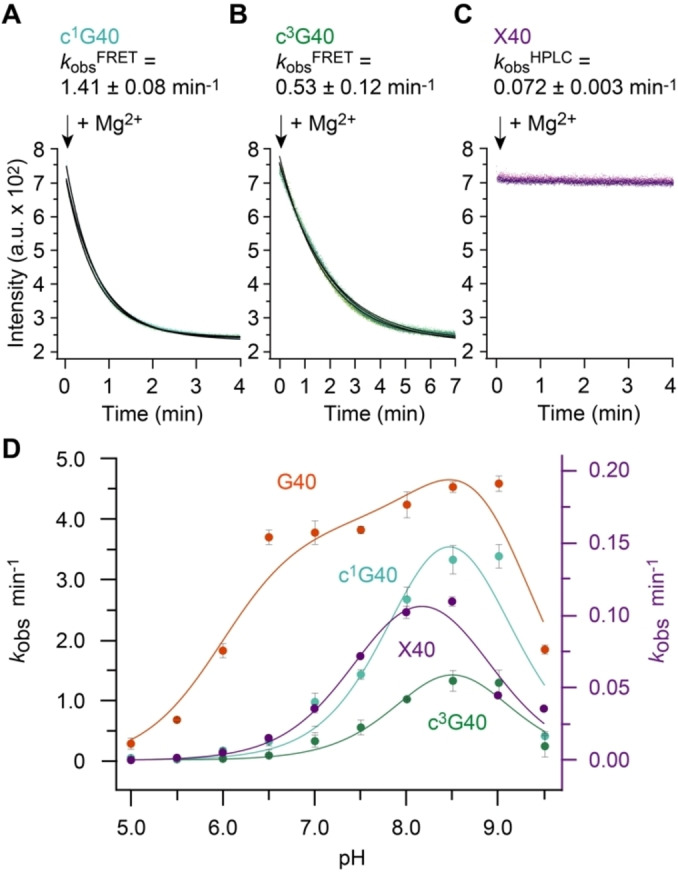
G40 mutants of pistol ribozyme. Exemplary set of FRET responses for A) c^1^G40, B) c^3^G40, and C) X pistol mutants followed by FRET. D) Activity‐pH profiles for wildtype and mutant pistol ribozymes. Note that *k*_obs_ values for the slow cleaving X‐mutant were determined by an HPLC assay (see Supporting Information). Conditions: *c*(RNA)=0.5 μM of each RNA strand (1 : 1 ratio); 10 mM MgCl_2_, 100 mM KCl, 50 mM MOPS, pH 7.5, 25 °C.

We thought that the most meaningful functional verification would be atomic mutagenesis of the N1 atom by replacement with a carbon atom (Figure 4C), which consequently deletes acid/base properties of this very position. According to a recent review, such an approach would concern a primary γ‐effect and it has been referred to as “general base knockout”.^[41]^ We therefore synthesized the c^1^G40 containing pistol mutant needed here. Figure 5 illustrates the result of c^1^G40 pistol ribozyme on backbone cleavage. The cleavage rate was only reduced by a factor of 2.7 (*k*_obs_ 1.41 min^−1^) compared to the wildtype G40 ribozyme (Table 1). This is indeed surprising because in the general view, the guanine N1‐H moiety activates the 2′‐OH either by formation of a hydrogen bond to the 2′‐OH nucleophile,^[25b]^ thus increasing its acidity, or the environment in the active site pocket shifts the p*K*_a_ towards neutrality so that the deprotonated guanine accepts the proton of the attacking 2′‐OH (concept of “reverse protonation”, see above). Clearly, c^1^G does not offer these functionalities and therefore, a much higher impact on activity was expected upon N1‐by‐C1 replacement. In this context, we note that the p*K*_a_ of c^1^G is 9.1,^[37]^ attributed to the phenolic OH. This p*K*_a_ value is comparable to the p*K*_a_ of G and it is tempting to speculate that the deprotonated O6 in c^1^G may take over the role of a deprotonated N1 in G although it is dislocated compared to the N1 position. We therefore tested a pistol mutant with 1‐deazainosine (c^1^I40; Figure 4C) that only provides the phenolic O6 at the Watson–Crick face and observed a 92‐fold decrease in cleavage rate (Table 1) which is not supportive for this speculation. At this point we note that for inosine at position 40, a decrease in cleavage rates was also observed as reported earlier (Supporting Table S3).[^32^, ^33^]

**Table 1:** Pistol ribozyme cleavage kinetics (*k*_obs_) and p*K*_a_ analysis from activity‐pH profiles (shown in Figure 5).^[a]^

Pistol variant	*k*_obs_ (pH 7.5)^[b]^ [min^−1^]	p*K*_a_ (N1‐H or O6‐H of nucleotide‐40) general base	p*K*_a_ (H_2_O‐Mg^2+^ coordinated to N7 of G33) general acid^[c]^	p*K*_a_ (influencer)^[d]^	Δp*K*_a_^coop[d]^	p*K*_a_ (free nucleoside)
G40 (wt)	3.79±0.23	7.99±0.03	8.90±0.01	6.94±0.03	1.94±0.03	9.2 (N1)^[47][e]^
c^1^G40	1.41±0.08	8.13±0.01	8.81±0.10	–		9.1 (O6)^[37]^
c^3^G40	0.53±0.12	8.33±0.08	8.66±0.08	–		12.3 (N1)^[42]^
X40	0.072±0.003	7.58±0.18	8.76±0.19	–		5.7 (N3)^[46]^
c^1^I40	0.041±0.007	–	–	–		9.5 (O6)^[f]^

[a] Cleavage kinetics of wildtype, c^1^G and c^3^G pistol were measured using the FRET assay, cleavage kinetics of X40 and c^1^I40 modified pistol RNA was obtained via HPLC (Supporting Information). All FRET rates were independently evaluated and confirmed by HPLC assays. For full sets of HPLC and FRET traces see Supporting Figures S2 to S17. [b] Conditions: 10 mM MgCl_2_, 100 mM KCl, 50 mM MOPS, pH 7.5, 25 °C. [c] The hydrated Mg^2+^ in the active‐site is coordinated to N7 of G33, and observed in all crystal structures of pistol;[^30^, ^31^, ^32^, ^33^] the p*K*_a_ of free Mg(H_2_O)_6_^2+^ is 11.44±0.1.[^48^, ^49^] [d] For definition of “influencer” and Δp*K*_a_^coop^ see work of Bevilacqua[^22^, ^23^] and the main text. [e] For p*K*_a_ values of guanosine derivatives see Refs. [47, 50] and for guanine in context of short oligodeoxynucleotides see Refs. [51, 52]. [f] Determined for the free nucleobase by UV‐spectroscopic titration experiments (in 25 mM tris(hydroxymethyl)aminomethane (TRIS), 100 mM KCl).

### Higher p*K*_a_ of General Base (c^3^G40)

To further shed light on the role of G40 in pistol ribozyme cleavage, we synthesized the c^3^G40 mutated version. The c^3^G modification provides the identical Watson–Crick—face as native guanosine and thus provides the same donor‐acceptor pattern for interactions with the attacking 2′‐OH nucleophile and with the transition state. However, the p*K*_a_ of c^3^G (free nucleoside) is 12.3[^36^, ^42^] and therefore significantly higher compared to G (p*K*_a_ 9.2). This certainly affects H‐bonding properties and the propensity for activation of the 2′‐O nucleophile. Indeed, the c^3^G40 mutation had a larger impact on cleavage compared to c^1^G40, reflected in a 7.2‐fold decrease in the observed rate (Figure 5B). Nevertheless, a second aspect must be taken into account for c^3^G40 modified pistol, namely, the H‐bond interaction between N3‐G40 (wildtype) and H_2_N(C4) of C41 is impaired,[^30^, ^31^, ^32^] and thus, a less ordered active site may account for slower cleavage beside the contribution arising from the altered p*K*_a_.

### Lower p*K*_a_ and Electrostatic Repulsion of General Base (X40)

Next, we investigated the impact of xanthosine in position 40 on pistol activity. This mutation changes the H‐donor properties of the original exocyclic NH_2_ (for G) to H‐acceptor properties through the C2 carbonyl group (for X). Given the generally accepted opinion that in many nucleolytic ribozymes the C2‐NH_2_ group of the guanine general base is crucial for stabilizing the pentavalent phosphorane transition state by H‐bond interactions with one of the non‐bonding oxygens,[^43^, ^44^] the O2 in xanthine will cause electrostatic repulsion with the transition state. For pistol this expectation is underpinned by the distance between the exocyclic NH_2_ and the non‐bridging oxygen observed in the crystal structure of the transition state mimic (Figure 4A).^[24]^ Indeed, the X40 mutation had a significant impact on activity, reflected in a 53‐fold decrease in cleavage rate (Figure 5C). We further mention that free xanthosine has a p*K*_a_ of 5.7 (attributed to N3‐H, and not N1‐H!),[^45^, ^46^] and is largely deprotonated at physiological pH values, with a significantly negative charge distribution at the O2 atom.

### Apparent p*K*_a_ Values from Activity‐pH Profiles

To shed further light on the specific impact of G40 as general base in pistol ribozyme cleavage we recorded activity‐pH profiles of the wildtype and the diverse mutant ribozymes by additional experiments at pH values ranging from 5.0 to 9.5 (Figure 5D). This approach allows to trace the titration curves according to the general acid and general base, and provides the actual (usually shifted) p*K*_a_ values of the titratable functional groups in the catalytic pocket.

Our experiments for the wildtype pistol ribozyme revealed a “wavy” cleavage rate‐pH profile with two activity maxima, one around pH 7 and the other around pH 8.7. These findings strongly suggest a second cationic species, interacting with the general base and thus lowering its p*K*_a_. As reported previously by Bevilacqua and others, residues surrounding the catalytic center, such as nucleobases, the ribose‐phosphate backbone, metal ions and cofactors are able to assist as influencer, facilitating general acid base catalysis.[^22^, ^23^, ^53^] To further analyze cooperative interactions in the pistol ribozyme we determined the p*K*_a_ values by applying a cubic cooperative model (using the published equations)^[22]^ with a p*K*_a_ of 6.94 assigned to the influencer, 7.99 for the general base, a Δp*K* ^coop^ of 1.94 and 8.9 for the general acid (Table 1). Obviously, the predicted p*K*_a_ for the influencer remained slightly higher than observed in the data points (rounding point ≈6.5) whereas the p*K*_a_ values for the general base and acid are predicted marginally lower compared to the actual rounding points of the measured profile (>8 for the base and >9 for the acid). Further, the coupling Δp*K* ^coop^ between the influencer and the general base of 1.94 is predicted higher than observed in the first plateau, that extends only over 1 pH unit from 6.5 to 7.5. Nevertheless, we clearly see that the p*K*_a_ of the general base is significantly shifted to neutrality by Δp*K* ^coop^ units in the presence of an active protonated influencer species. Simultaneously, the activity of the influencer is enhanced by an upward shift of Δp*K* ^coop^ units towards the neutral range. Even in the absence of the functionally active influencer above pH 8, p*K*_a_ shifts for the general base of about 1.2 p*K*_a_ units and about 2 p*K*_a_ units for the general acid are observed compared to the unshifted values (9.2^[47]^ and 11.4,^[48]^ respectively).

In general, these observations can be attributed to the local electronic environment surrounding the catalytic residues, enhancing their catalytic activities.[^22^, ^23^, ^53^, ^55^] More precisely, for pistol, the nature of the cationic influencer can be associated most likely with the electrostatic potential of the “second” hydrated Mg^2+^ ion that is located near G40, observed in all crystal structures of precatalytic, transition state mimic, and product states.^[33]^ At this point we mention that also several computational studies shed light on the possible influencer activities,[^56^, ^57^, ^58^] one of them especially points out that the Hoogsteen edge of G40 is exposed to solvent in an electronegative pocket, making the O6 position available to (transiently) interact with metal ions (considerably more than other guanine residues) and that this can tune the p*K*_a_ at the N1 position.^[56]^ In another computational study, the interesting finding was made (by means of classical MD simulations and QM/MM hybrid calculations) that only the model where the protonation of the nucleotide base was according to the canonical state renders reactive conformations of the active site.^[57]^ This was not the case for the models in which guanine G40 was parametrized as deprotonated at the N1 atom (“reversed protonation”).^[58]^

Interestingly, the G40 variants tested here (c^1^G, c^3^G and X) lose the wavy shape of their rate‐pH profiles. They seem to lack cooperative interactions with an influencer, resulting in p*K*_a_ values in the same range as modelled for the wildtype in absence of an influencer. Finally, we note that also for the pistol ribozyme from *Paenibacillus polymyxa*, a “wavy” rate‐pH profile was observed although this feature was not further discussed in that study.^[59]^

### Short Note on the Role of G42

In a recent computational study, a possible catalytic role of an alternative conserved guanosine in the binding pocket, namely G42, has been postulated.^[60]^ Under the assumption of a local rearrangement (into a L‐platform/L‐scaffold framework) simulated by molecular dynamics calculations, G42 has been proposed to act as general base in a primary catalytic pathway.^[60]^ Having the c^1^G modification in hands, we set out to experimentally test this hypothesis with the corresponding G42c^1^G mutant. We found that the cleavage rate for the mutated ribozyme was 0.89±0.06 min^−1^ which is only 4.2‐fold slower compared to the wildtype ribozyme (Supporting Figure S11, Supporting Table S3). Therefore, even if G42 would be able to take over the role of G40, its contribution to catalysis is minor. We further note that G42 bridges G40 and A32 via an H‐bond network (2.7 Å distance between A32 2′‐OH and the NH_2_ of G42; 2.7 Å distance between the O6 of G42 and the NH_2_ of G40) and thereby generates a cleft to accommodate the scissile phosphate of the splayed‐apart G53–U54 nucleosides (Supporting Figure S11A).[^30^, ^34^] The c^1^G mutation retains these interactions while other modifications, such as 2‐aminopurine (Ap), do not. The significant rate reduction previously observed for G42Ap mutated pistol ribozyme may arise from this structural impairment.^[32]^

## Conclusion

Nucleolytic ribozymes accelerate the cleavage reaction by more than a million‐fold (10^7^ to 10^8^).^[8]^ They seem to apply the four strategies α, β, γ‐, and δ (Figure 1) to different extent, however, for most of the ribozyme classes the weighting of the different contributions is unclear. One reason for this is that experimental means, i.e. RNA atomic mutagenesis of the functionality that is responsible for guanosine acid‐base catalysis (namely, the N1‐H moiety of guanine) have been lacking until recently.^[37]^

**Figure 6:**
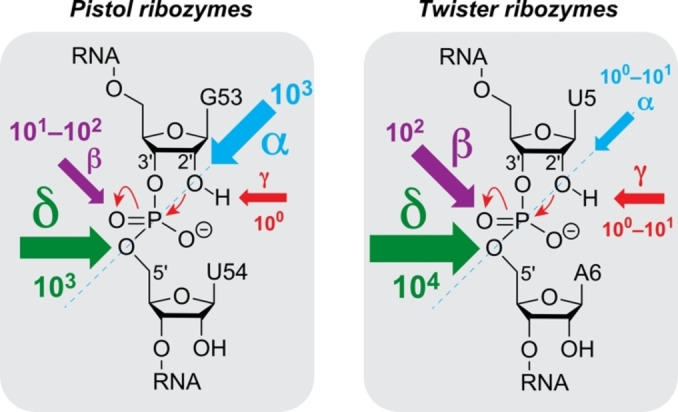
Scaling catalytic contributions for small self‐cleaving ribozymes. General base knockout through c^1^G mutagenesis together with data form earlier mutagenesis studies reveal α‐ and δ‐catalysis as major determinant for the pistol ribozyme class while β‐ and δ‐catalysis is dominant for the class of twister ribozymes. Weighting is illustrated by the size of arrows and the estimate of rate enhancement by the order of magnitude as indicated. See main text for details and explanation of α, β, γ, δ factors.

The present study provides scaling of catalytic contributions for the pistol ribozyme class with unprecedented precision. Although it is tempting to assume that in general γ‐ and δ‐catalysis—jointly constituting concerted general acid‐base catalysis—make the largest contribution to the catalytic rate enhancement,^[8]^ for pistol ribozymes this is not the case. Clearly, retirement of the guanine N1 functionality by atomic mutagenesis through a carbon atom erases the acid‐base properties at the very position. Despite this fact, the rate is only reduced by a factor of 2.7, and hence, γ‐catalysis is a subordinate player in pistol catalysis (Figure 6). As we previously demonstrated, a major contribution for pistol activity originates from δ‐catalysis. When the coordination site N7 of G33 for hydrated Mg^2+^ was mutated to C7 (c^7^G33), the cleavage rate was reduced by an order of at least 10^3^.[^32^, ^33^, ^34^] Therefore, δ‐catalysis executed by the N7‐G33 innersphere coordinated, hydrated Mg^2+^ as general acid provides a significantly higher contribution compared to γ‐catalysis of the general base G40. The question still is where the remaining factor of about 10^3^ to 10^4^ to achieve an overall acceleration of 10^7^ to 10^8^ originates from. This acceleration apparently stems from α‐ and β‐catalysis. For pistol, the contribution through transition state stabilization (β‐catalysis) is also small as deduced from comparatively small rate reductions by c^3^G (6‐fold), xanthosine (53‐fold), and 1‐deazainosine (92‐fold; note that c^1^I lacks the possibility for bidendate interaction with the transition state while the other two mutants retain at least one possibility for strong H‐bonding; see Figure 4A, C). Hence, the remaining major contribution of about 10^3^ must originate from α‐catalysis, meaning proper in‐line alignment of the attacking 2′‐O nucleophile to the to‐be‐cleaved P O5′, ideally with an angle *τ* of 180° (*τ* describes the angle O2′ (of nucleotide N−1) to P−O5′ (of nucleotide N+1) according to Ref. [9]). Support for a dominant role of positioning comes from crystallography. All structures of the pre‐catalytic pistol fold from three independent laboratories reveal an angle *τ* close to 180°. Additionally, our FRET folding assay indicates that the extended conformation of the substrate is already adopted prior attack of the nucleophile at the scissile phosphate. Notably, given the prominent role of substrate positioning in the ribosome and of cofactor positioning in recently found methyltransferase ribozymes,[^61^, ^62^] the feature of “positioning” accommodates perfectly into this argumentation for the pistol ribozymes.

Finally, we note that the strict phylogenetic conservation of pistol G40 still makes sense from the point of evolution, aiming at utmost activity attainable from the four naturally available nucleotides. Pistol mutants G40A, G40C, and G40U have 34‐fold, 94‐fold, and 152‐fold less cleavage activity (Supporting Table S3, Supporting Figures S12–S14). This is consistent with the small contribution of β‐ and γ‐catalysis of nucleotide‐40 (10^1^–10^2^) to the estimated overall rate enhancement in pistol ribozymes. Even G40rS (lacking the nucleobase completely) and the double mutant G40c^1^G–G42c^1^G have comparable residual cleavage activities (60‐fold and 49‐fold decrease, respectively) (Supporting Table S3, Supporting Figures S15 and S17).

To date, only one other ribozyme class has been scaled for catalytic contributions with high precision. This is twister where “general base knockout” using c^1^G atomic mutagenesis caused a 275‐fold reduction in cleavage rate.^[37]^ The precatalytic crystal structure of twister shows a hydrogen bond (2.6 Å) between the N1 atom of the active site guanine and the nonbridging *pro*‐R oxygen of the scissile phosphate,^[63]^ and hence, implicates that this guanine (G48) plays a significant role in phosphorane transition state stabilization (β‐catalysis). At the same time, a more prominent role in activation of the 2′‐OH (γ‐catalysis) cannot be excluded for this guanine. In contrast to pistol, for twister ribozymes, α‐catalysis is small. It was demonstrated that a single, conformationally flexible nucleoside directly 5′ of the scissile phosphate is readily cleaved.^[64]^ Moreover, several more crystal structures of the precatalytic twister caught the nucleoside 5′ of the scissile phosphate in different conformations (with angles *τ* ranging from 83 and 148°),^[26]^ consistent with hardly any pre‐organization/pre‐folding of this nucleoside in the twister active site.

In summary, our study demonstrates a reliable approach for scaling the different contributions of catalysis (α, β, γ, δ) in individual self‐cleaving ribozymes. This becomes possible by directly addressing the key functionality N1 of the general base guanine through atomic mutagenesis with c^1^G. This feature of a “general base knockout” has been neglected in earlier mechanistic studies of nucleolytic ribozymes. Our study paves the way for scaling catalytic factors in other ribozymes as well, and therefore, contributes to a thorough mechanistic understanding of ribozyme catalysis.

## Conflict of interest

The authors declare no conflict of interest.

1

## Supporting information

As a service to our authors and readers, this journal provides supporting information supplied by the authors. Such materials are peer reviewed and may be re‐organized for online delivery, but are not copy‐edited or typeset. Technical support issues arising from supporting information (other than missing files) should be addressed to the authors.

Supporting Information

## Data Availability

The data that support the findings of this study are available from the corresponding author upon reasonable request.
