# Supplementary material for: Scaling Catalytic Contributions of Small Self‐Cleaving Ribozymes
Source: Angew Chem Int Ed Engl. 2022 Sep 2;61(41):e202207590. doi: 10.1002/anie.202207590 (PMC9826390; doi:10.1002/anie.202207590)
Supplement: Supplementary file 1 — Supporting Information [file ANIE-61-0-s001.pdf]

Angewandte Chemie
Eine Zeitschrift der Gesellschaft Deutscher Chemiker

Supporting Information

Scaling Catalytic Contributions of Small Self-Cleaving Ribozymes

*M. Egger, R. Bereiter, S. Mair, R. Micura**

Supporting Information

Contents

Supporting Methods

2'-*O*-(3'-Azidopropyl)uridine modified solid support S02
RNA solid-phase synthesis, deprotection, and purification S12
Mass spectrometry S13
Preparation of double labeled RNA by N-hydroxysuccinimide ester (NHS) chemistry and strain-promoted alkyne-azide cyloadditions (SPAAC) S13
Ribozyme cleavage (HPLC assay) S14
Ribozyme cleavage (FRET assay) S14
Determination of pH - rate profiles for ribozyme cleavage S14

Supporting Figures

Supporting Figure S1 S16
Supporting Figure S2 S17
Supporting Figure S3 S18
Supporting Figure S4 S19
Supporting Figure S5 S20
Supporting Figure S6 S21
Supporting Figure S7 S22
Supporting Figure S8 S23
Supporting Figure S9 S24
Supporting Figure S10 S25
Supporting Figure S11 S26
Supporting Figure S12 S27
Supporting Figure S13 S28
Supporting Figure S14 S29
Supporting Figure S15 S30
Supporting Figure S16 S31
Supporting Figure S17 S32

Supporting Tables

Supporting Table S1 S33
Supporting Table S2 S34
Supporting Table S3 S35

References S36

Synthesis of 2'-*O*-(3-azidopropyl)uridine modified solid support[1]

In analogy to: Santner, T., Hartl M., Bister K. and Micura R. (2014). *Bioconjugate Chem.*, 25, 188-95.

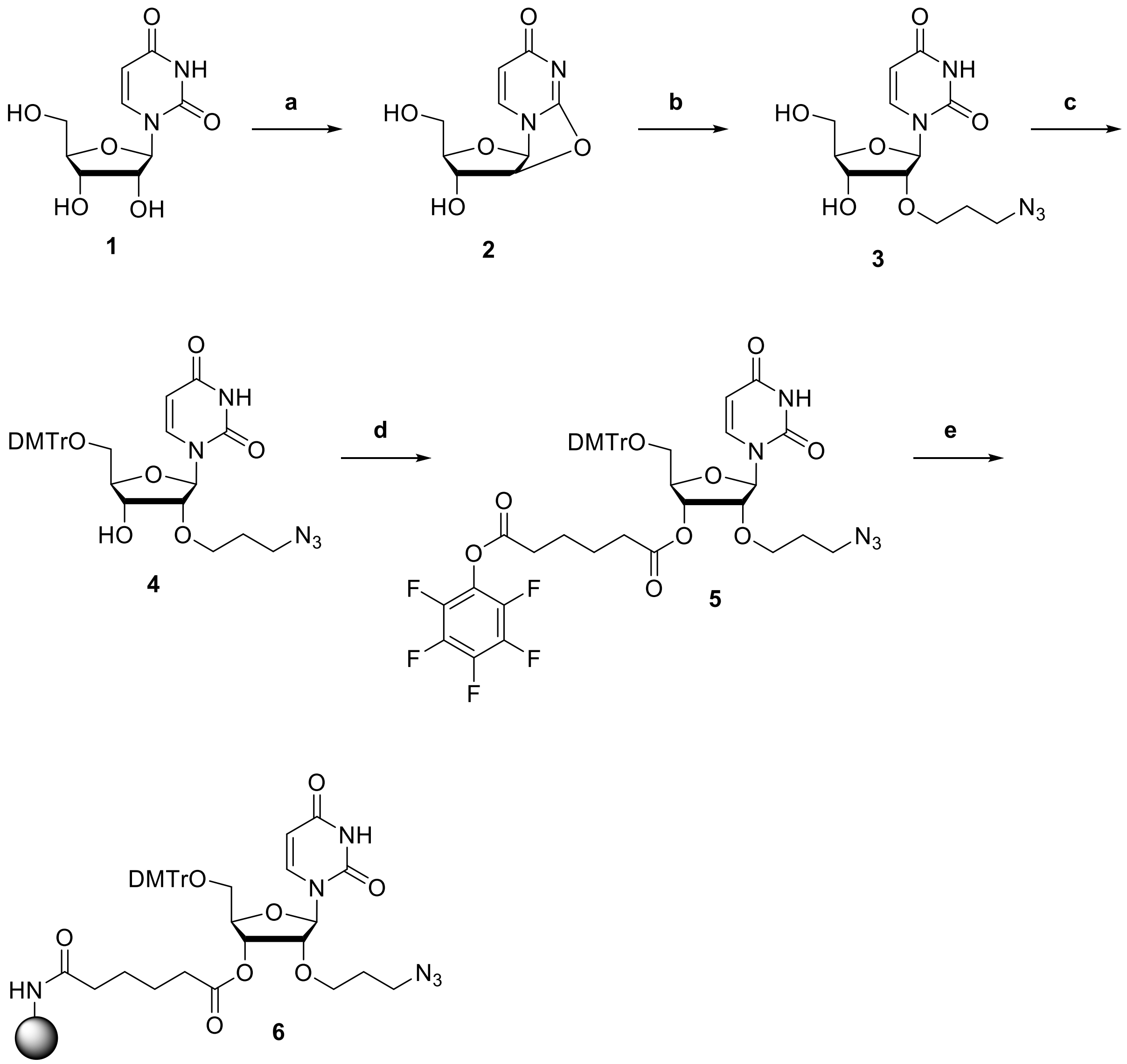

Reaction conditions: **a.** 1.2 eq. Diphenylcarbonate, 0.08 eq. $NaHCO_3$, DMF, 110 °C, 4 h, 81%; **b.** 5 eq. Azidopropanol, 3 eq. BF_3•OEt_2, DMA 120 °C, 16 h, 38% of **3**; **c.** 1.1 eq. DMTCl, pyridine, 16 h, r.t., 81% of **4**; **d.** 3 eq Adipicacidpenta-fluorophenylester, 1.2 eq DMAP, DMF/py (1:1), 2 h, r.t., 44% of **5**; **e.** amino functionalized solid support, DMF, 48 h, r.t., loading density of **6**: 40 μmol/g.

2,2'-*O*-Anhydro(*β*-D-arabinofuranosyl)uracil (2)

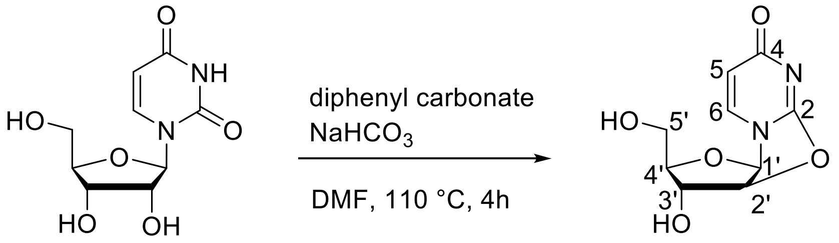

[244.20] [226.19]

$C_9H_{12}N_2O_6$ $C_9H_{10}N_2O_5$

1 **2**

Diphenyl carbonate (4.21 g, 19.7 mmol, 1.2 eq) was suspended in *N,N*-dimethylformamide (5.5 ml) and uridine (4.00 g, 16.4 mmol, 1 eq) was added. The suspension was heated to 80 °C and sodium bicarbonate (110 mg, 1.3 mmol, 0.08 eq) was added. The reaction mixture was stirred at 110 °C for 4 hours. Gas evolution was observed and the product precipitated from the clear solution. After cooling to room temperature, the precipitate was collected by filtration and was washed with methanol and dried.
Yield: 2.99 g (81%), white solid

TLC (methanol/dichloromethane, 15:85): *Rf* = 0.2

^{1}H-NMR (400 MHz, d6-DMSO): δ 3.19 – 3.28 (m, 2H, H_2-C(5′)); 4.07 (m, 1H, H-C(4′)); 4.39 (1H, H-C(3′)); 4.98 (1H, H-O(5′)); 5.20 (d, J = 5.17 Hz, 1H, H-C(2′)); 5.84 (d, J = 7.48 Hz, 1H, H-C(5)); 5.88 (1H, H-O(3′)); 6.30 (d, J = 4.80, 1H, H-C(1′)); 6.72 (d, J = 7.40 Hz, 1H, H-C(6)) ppm.

^{13}C NMR (100 MHz, d6-DMSO): δ 61.60 (C(5′)); 75.50 (C(3″)); 89.20 (C(2′)); 89.78 (C(4′)); 90.50 (C(1′); 81.93 (C(2′); 109.70 (C(5)); 137.40 (C(6)) ppm.

^{1}H NMR (400 MHz, d6-DMSO)

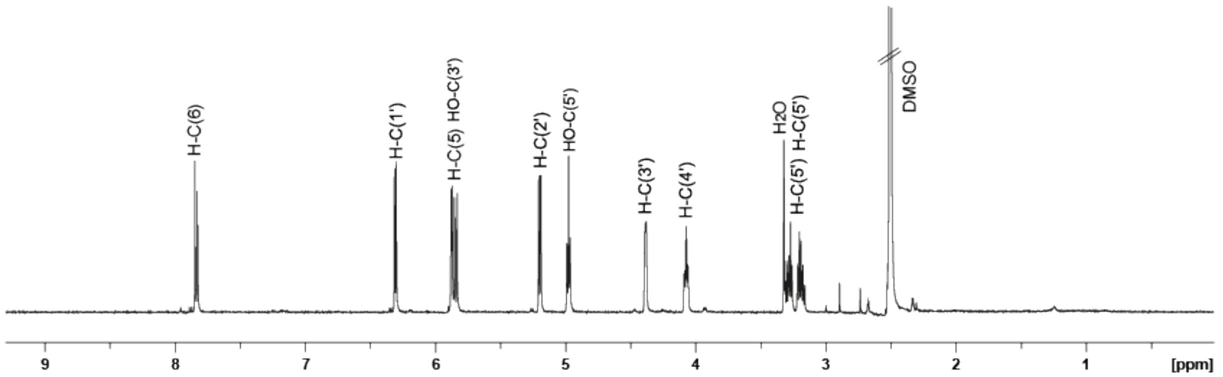

^{13}C-NMR (100 MHz, d_6-DMSO)

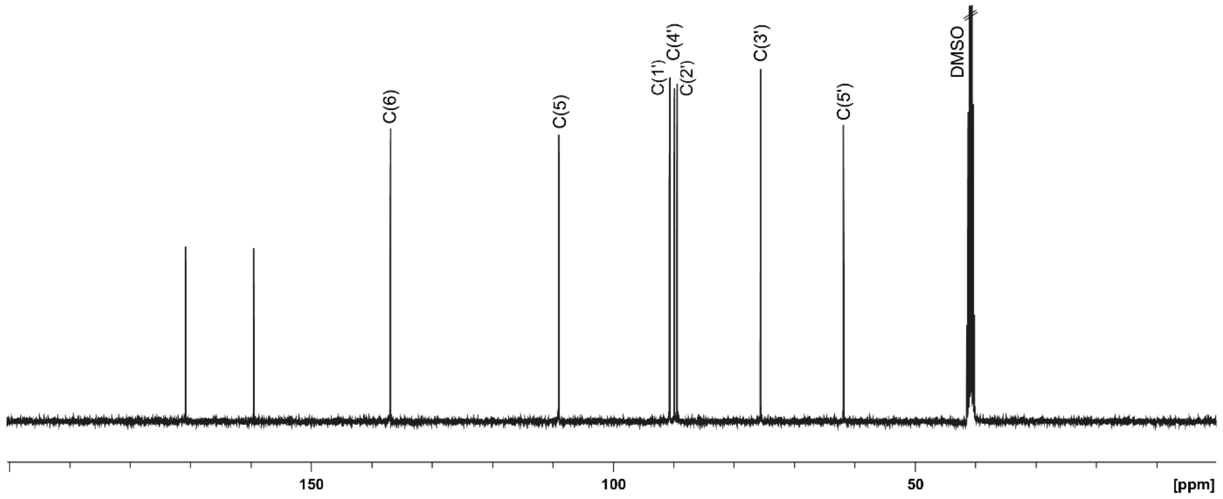

2'-(3''-Azidopropoyl)uridine (3)

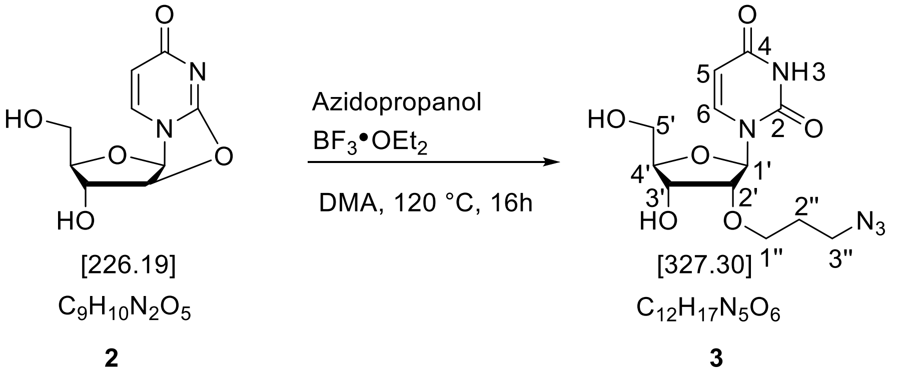

Prior to the reaction compound 2 (568 mg, 2.51 mmol) was coevaporated three times with dry pyridine and was stored over phosphorous pentoxide (P_2O_5) in a desiccator overnight. It was suspended in (4ml) N,N-dimethylacetamide and azidopropanol (1.35 ml, 14.58 mmol, 5.8 eq) as well as boron trifluoride diethyl etherate (1.27 ml, 10.05 mmol, 4 eq) were added. The reaction mixture was heated to 120 °C for 16 hours. All solvents were removed in vacuo and the remaining residue was absorbed on silica in methanol. Purification of the crude product was performed by silica gel column chromatography (methanol/dichloromethane, 0:100-3:97). **Yield:** 319 mg (38%), white foam

TLC (methanol/dichloromethane, 15:85): *Rf* = 0.55

^{1}H NMR (400 MHz, d6-DMSO): δ 1.78 (m, 2H, H_2−C(2″)); 3.41 (m, 2H, H_2−C(3″)); 3.60 (m, 4H; H_2−C(5′), H_2−C(1″)); 3.88 (m, 2H, H−C(2′), H−C(4′)); 4.09 (dd, J = 5.49 Hz, J = 10.33 Hz, 1H, H−C(3′)); 5.09 (d, 1H, J = 6.29 Hz, HO-C(3′); 5.14 (t, 1H, J = 5.16 Hz, HO-C(5′); 5.64(d, J = 8.94 Hz, 1H, H−C(5)); 5.87 (d, J = 4.48 Hz, 1H, H−C(1′)); 7.93 (d, J = 8.18 Hz, 1H, H−C(6)); 11.33 (s, 1H, NH)ppm.

^{13}C NMR (100 MHz, d6-DMSO): δ 29.20 (C(2″)); 48.50 (C(3″)); 61.02 (C(5′)); 67.22 (C(1″)); 68.91 (C(3′); 81.93 (C(2′); 84.93 (C(4′)); 85.37 (C(1′)); 102.48 (C(5)); 140.81 (C(6)); 151.21; 163.80 ppm.

^{1}H NMR (400 MHz, d6-DMSO)

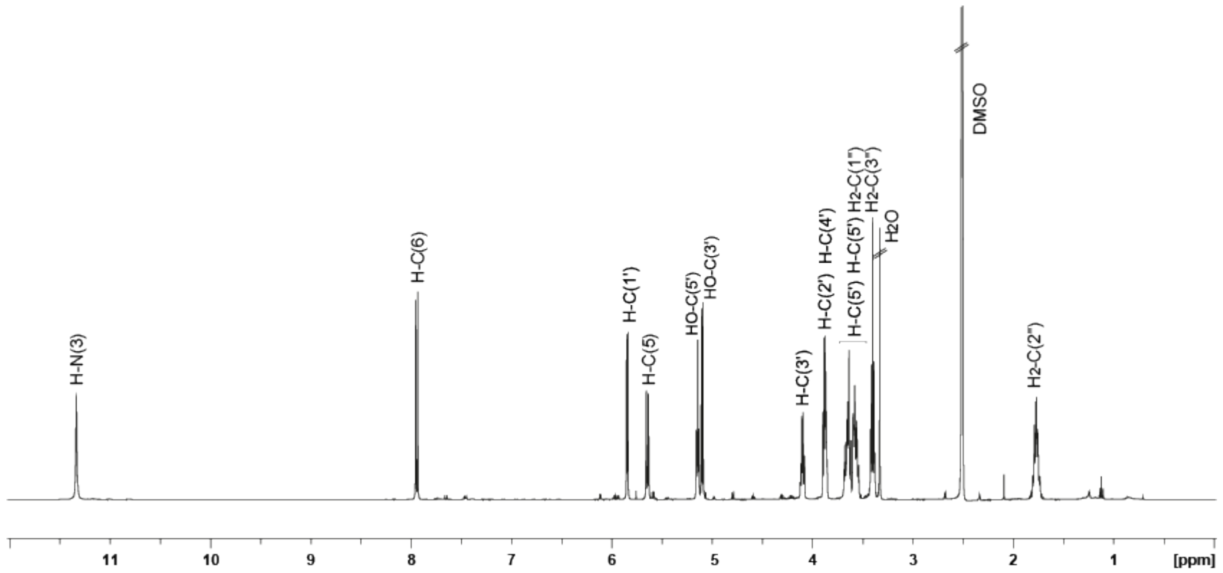

^{13}C-NMR (100 MHz, d_6-DMSO)

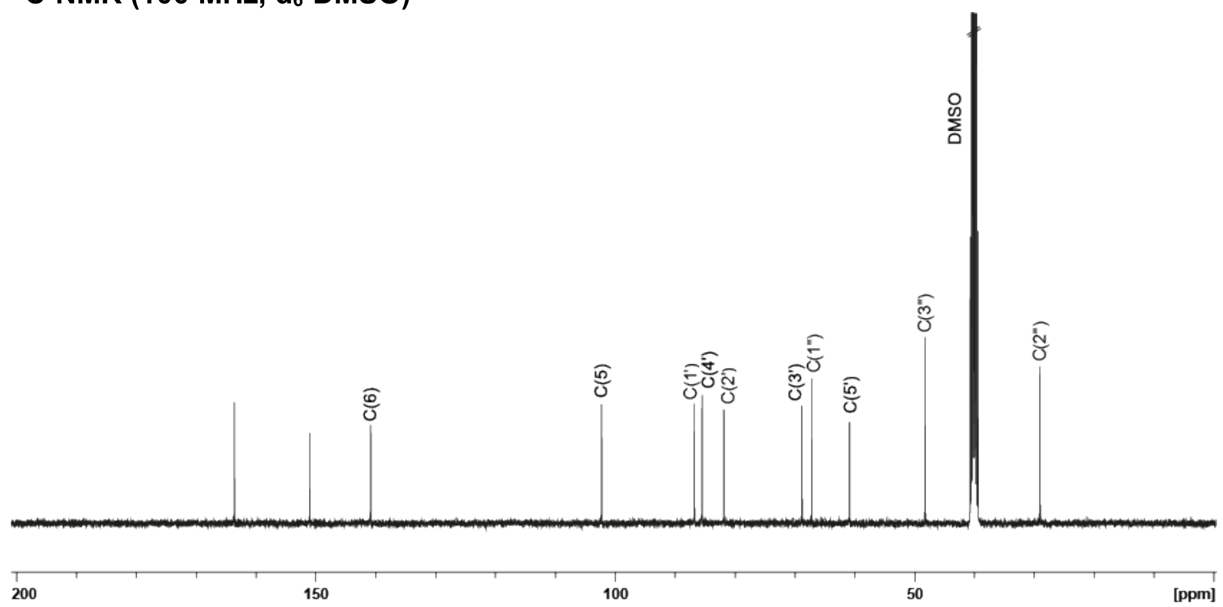

2'-*O*-(2-Azidopropyl)-5'-*O*-(4,4'-dimethoxytrityl)uridine (4)

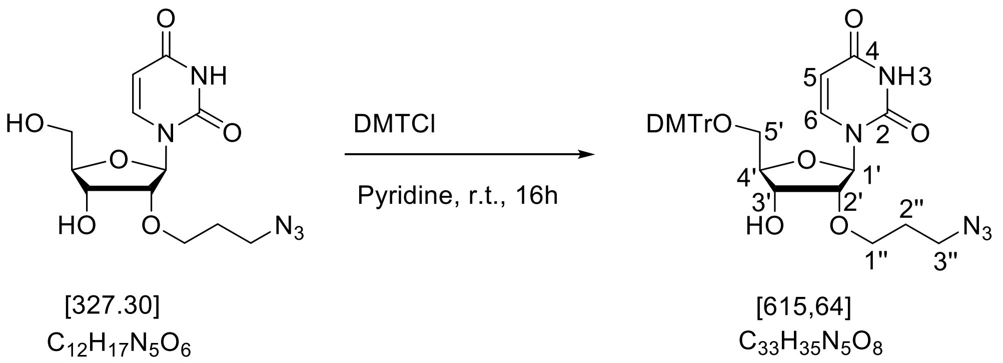

3 **4**

Compound 3 (148 mg, 452 µmol) was coevaporated three times with anhydrous pyridine and dried on high vacuum overnight. The solid was dissolved in 4.5 ml pyridine and 4,4'-Dimethoxytrityl chloride (168 mg, 497µmol) was added portion wise within 1.5 hours. The solution was stirred at room temperature overnight until the reaction was complete, monitored by TLC. The reaction was quenched with methanol and reduced in vacuo. The residue was diluted with dichloromethane and extracted with 5% citric acid, water and saturated $NaHCO_3$. The crude product was purified by silica gel column chromatography (methanol/ dichloromethane, 0:100-2:98).
Yield: 227 mg (80%), white foam

TLC (methanol/dichloromethane, 15:85): *Rf* = 0.70

^{1}H NMR (400 MHz, d6-DMSO): δ 1.82 (m, 2H, H_2−C(2″)); 3.28 (m, 2H, H−C(5′)); 3.44 (m, 2H, H_2−C(3″)); 3.68 (m, 2H; H_2−C(1″)); 3.74 (s, 6H, H_3-C-O), 3.94 (m, 1H, H−C(2′)); 3.98 (m, 1H, H−C(4′)); 4.20 (dd, 1H, J = 12.34 Hz, J = 6.43 Hz, H-C(3′); 5.17 (d, 1H, J = 6.55 Hz, HO-C(3'); 5.29(d, J = 8.49 Hz, 1H, H−C(5)); 5.80 (d, J = 3.09 Hz, 1H, H−C(1′)); 6.90 – 6.93 (d, 4H, J =8.54, H-C(ar)); 7.26-7.39 (m, 9H, H-C(ar)); 7.73 (d, J = 7.85 Hz, 1H, H−C(6)); 11.38 (s, 1H, NH) ppm.

^{13}C NMR (100 MHz, d6-DMSO): δ 29.20 (C(2″)); 48.30 (C(3″)); 55.81 (2C, C(O-CH_3)); 63.35 (C(5′)); 67.42 (C(1″)); 69.11 (C(3′); 81.56 (C(2′); 83.02 (C(4′)); 86.52; 87.64 (C(1′)); 102.17 (C(5)); 113.74 (4C, C(ar)); 127.33-130.46 (9C, C(ar)); 135.65; 140.84 (C(6)),145.70; 150.36; 158.84; 163.80 ppm.

^{1}H NMR (400 MHz, d6-DMSO)

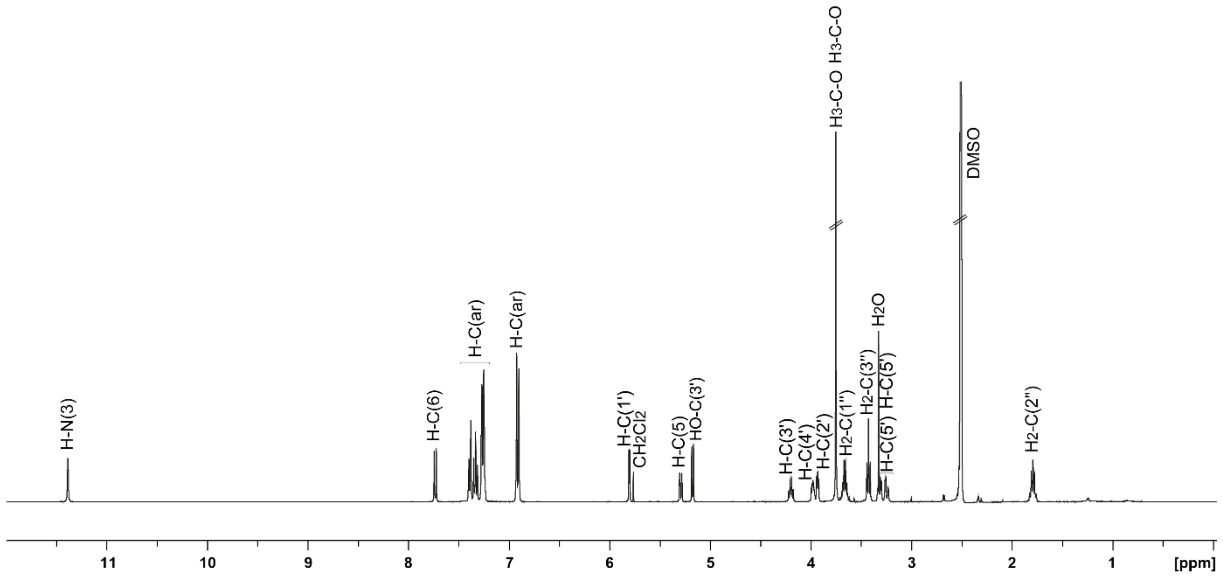

^{13}C-NMR (100 MHz, d_6-DMSO)

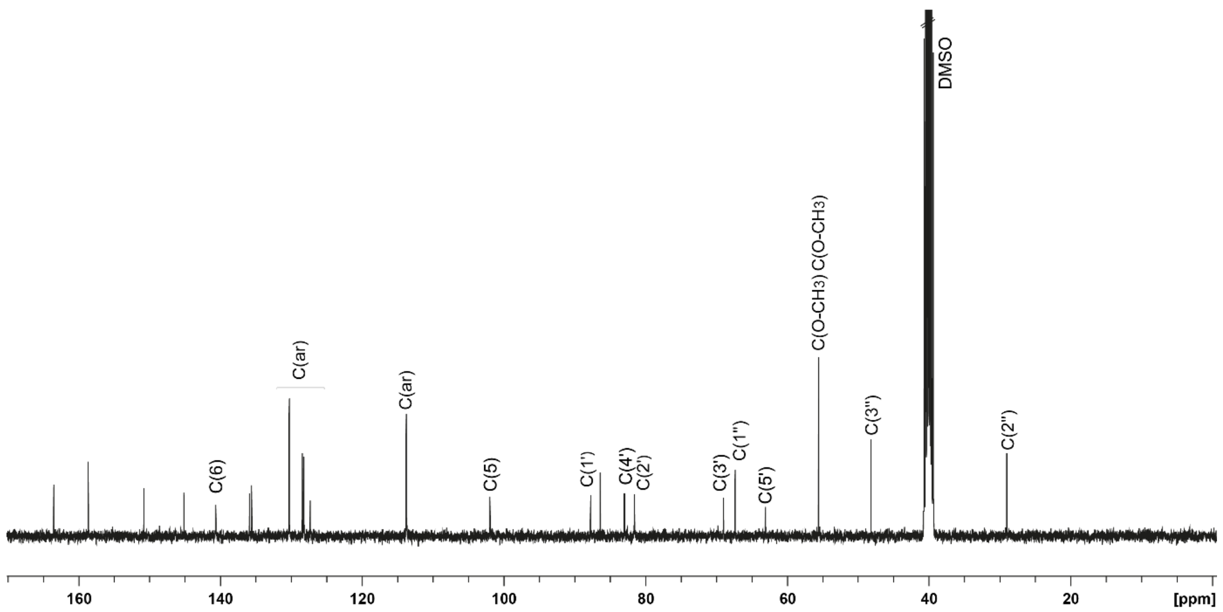

2'-*O*-(2-Azidopropyl)-5'-*O*-(4,4'-dimethoxytrityl)-3'-O-[1,6-dioxo-6-(pentafluorophenyl-oxy)hexyl]uridine (5)

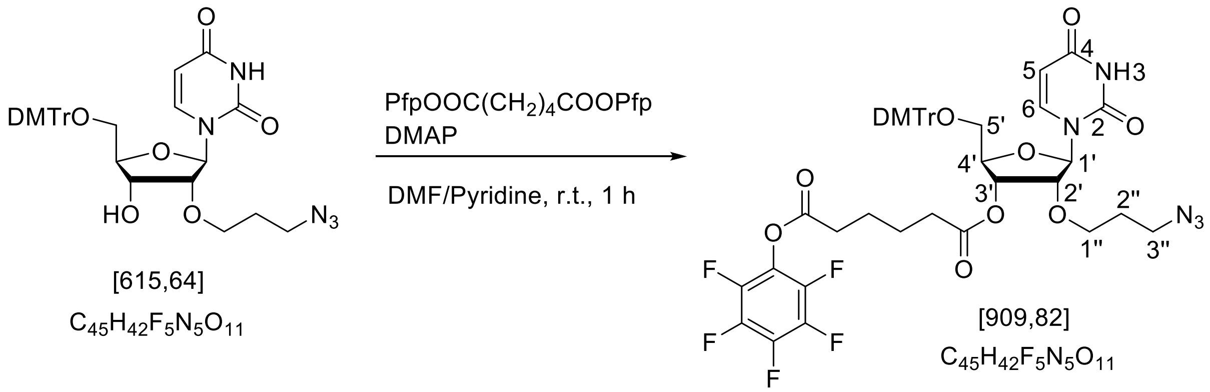

4 **5**

Compound 4 (162 mg, 258 µmol) was coevaporated three times with anhydrous pyridine and dried over P_2O_5 overnight. A mixture of anhydrous DMF/pyridine (1:1, 7.4 ml) was added, followed by the addition of 4-(dimethylamino)pyridine (37 mg, 309 µmol) and adipinic acid pentafluorophenyl ester 8443 mg, 927 µmol). The reaction mixture was stirred at room temperature for 1 hour. Then, the solvents were evaporated and the remaining liquid was coevaporated with acetone and dichloromethane. The The crude product was purified by silica gel column chromatography (acetone/dichloromethane, 1:99-5:95).

Yield: 108 mg (45%), white foam

TLC (acetone/dichloromethane, 20:80): *Rf* = 0.55

^{1}H NMR (400 MHz, $CDCl_3$): δ 1.74 (m, 6H, H_2−C(2″); RO_2-CH_2-$(CH_2)_2$-CH_2-CO_2-C_6F_5); 2.37 (m, 2H, RO_2-CH_2-$(CH_2)_2$-CH_2-CO_2-C_6F_5); 2.64 (t, J = 6.76 Hz, 2H, RO_2-CH_2-$(CH_2)_2$-CH_2-CO_2-C_6F_5); 3.28 (t, J = 6.76 Hz, 2H, H−C(3″)); 3.36 (dd, J = 11.33 Hz, J = 0.32 Hz 1H, H_2−C(5′)); 3.56 (m, 2H; H_2−C(1″), H_2−C(5′)); 3.68 (m, 1H, H−C1″)); 3.72 (s, 6H, H_3-C-O); 4.12 (t, J = 4.50 Hz, 1H, H−C(2′)); 4.18 (m, 1H, H−C(4′)); 5.18 (t, 1H, J = 4.28 Hz, H-C(3′); 5.26 (d, 1H, J = 6.26 Hz, H-C(5)); 5.93 (d, J = 4.27 Hz, 1H, H−C(1′)); 6.76 – 6.78 (d, 4H, J =9.98, H-C(ar)); 7.17 - 7.28 (m, 9H, H-C(ar)), 7.82 (d, J = 8.56 Hz, 1H, H−C(6)); 8.25 (s, 1H, NH) ppm.

^{13}C NMR (100 MHz, d6-DMSO): δ 24.21 (2C, RO_2-CH_2-$(CH_2)_2$-CH_2-CO_2-C_6F_5)); 29.24 (C(2″)); 33.02 (1C, RO_2-CH_2-$(CH_2)_2$-CH_2-CO_2-C_6F_5)); 33.75 (1C, RO_2-CH_2-$(CH_2)_2$-CH_2-CO_2-C_6F_5)); 48.16 (C(3″)); 55.33 (2C, C(O-CH_3)); 61.98 (C(5′)), 68.23 (C(1″)); 70.81 (C(3′); 81.44 (2C, C(2′), C(4′)); 87.95 (C(1′)); 102.57 (C(5)); 113.69 (4C, C(ar)); 127.58-130.36 (9C, C(ar)); 135.03; 140.02; 144.37 (C(6)); 145.70; 150.21; 159.19; 162.94; 172.07 ppm.

^{1}H NMR (400 MHz, d6-DMSO)

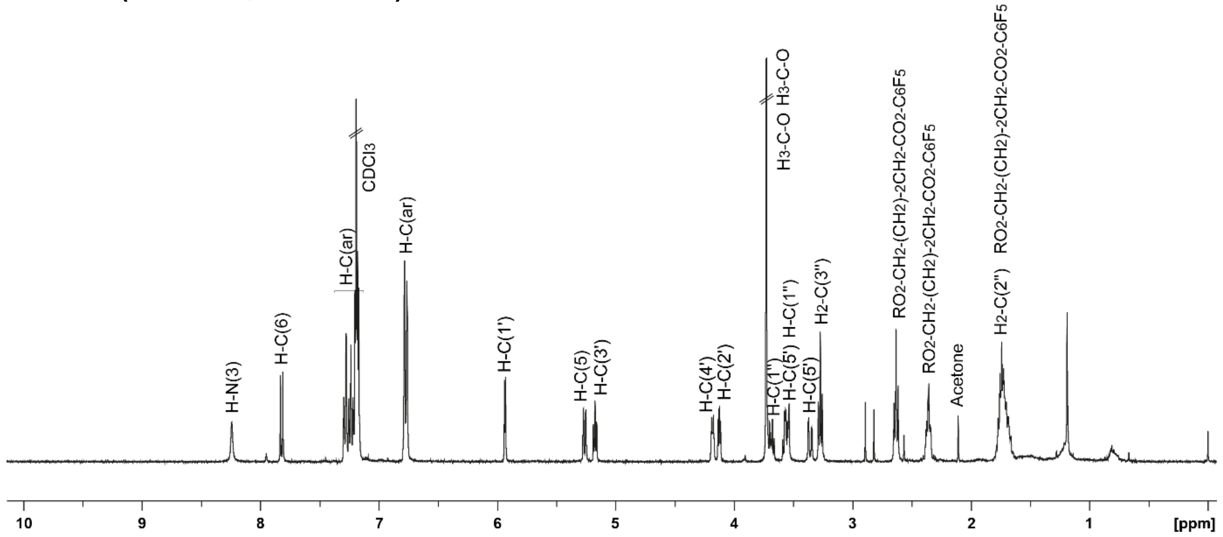

^{13}C-NMR (100 MHz, d$_6$-DMSO)

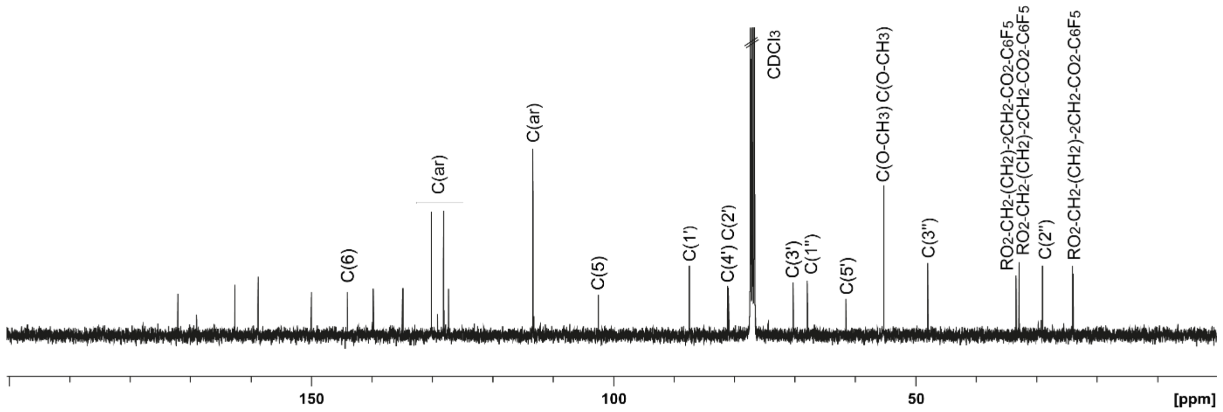

2'-*O*-(2-Azidoprpyl)uridine modified solid support (6)

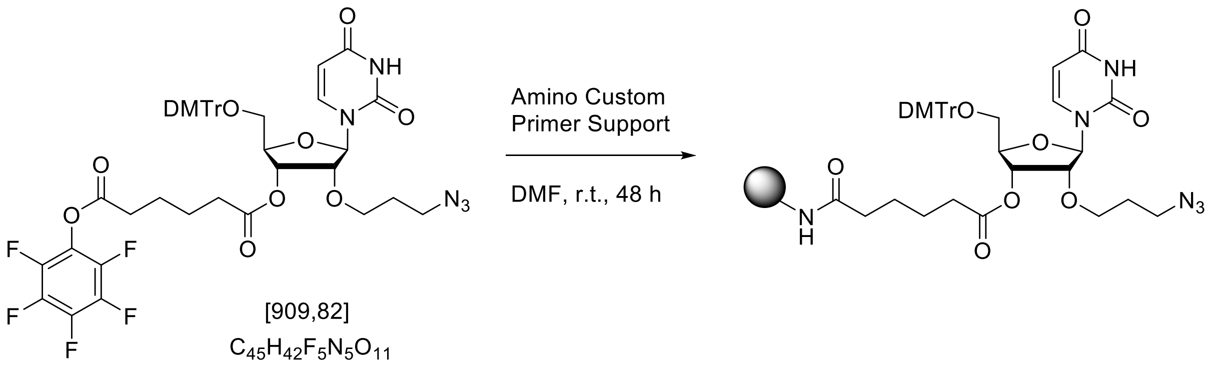

5 **6**

Amino-functionalized solid support (GE Healthcare, Custom Primer Support 200 Amino, 436 mg) was transferred into a syringe equipped with a polypropylene filter. The resin was washed with dry dichloromethane, followed by dry *N,N*-dimethylformamide. Then, the solid support was suspended and swelled in 2.5 ml *N,N*-dimethylformamide for 30 minutes.

Compound 5 (103 mg, 112 µmol) was dissolved in a small amount of *N,N*-dimethylformamide. Subsequently the mixture was combined with the resin suspension in the syringe. The suspension was shaken for 48 hours at room temperature. Then, the solvent was filtered off by the syringe and the remaining solid support was washed four times with *N,N*-dimethylformamide, methanol and dichloromethane, and subsequently allowed to dry. In a final capping step, the resin was treated with a mixture of 3.0 mL *Cap A* ((acetic anhydride/2,4,6-trimethylpyridine/acetonitrile, 2/3/5) and 3.0 mL *Cap B* (4-(*N,N*-dimethylamino) pyridine/ acetonitrile, 0.5 M) and was shaken for 4 min at room temperature. Finally, the solid support was washed several times with acetonitrile, methanol and dichloromethane. The product was removed from the syringe and dried under vacuum.

Loading (Trityl assay): 40 µmol/g

RNA solid-phase synthesis, deprotection, and purification

All RNAs were assembled on an ABI392 synthesizer at 1 µmol scale using 2'-*O*-TOM nucleoside phosphoramidites (ChemGenes), 5'-*O*-dimethoxytrityl-1'-deoxyribose-2'-*O*-TBDMS-3'-[(2-cyanoethyl)-(*N*,*N*-diisopropyl)]-phosphoramidite (rS amidite; Glen Research), polystyrene supports (GE Healthcare, Custom Primer Support™, 80 µmol g^{-1}; PS 200 and Primer Support™ 5G, 300 µmol g^{-1}) and CPG supports (ChemGenes, CPG 1000 Å, 30-40 µmol g^{-1}).

Standard RNA synthesis cycle: 1) detritylation with dichloroacetic acid/1,2-dichloroethane (4/96) (120 s); 2) coupling with phosphoramidites in acetonitrile (0.1 M) and benzylthiotetrazole in acetonitrile (0.3 M) (180 s); 3) capping with Cap A: phenoxyacetic anhydride (0.2 M) in dry THF and Cap B: *N*-methyl imidazole and *sym*-collidine (0.2 M each) in dry THF (2 x 15 s, Cap A/Cap B, 1:1); 4) oxidation with iodine (20 mM) in tetrahydrofuran (THF)/pyridine/H_2O (40/1/9) (60 s).

In-house synthesized modified nucleoside building blocks of c^1G, c^3G, and X were incorporated using modified synthesis cycles with longer coupling times (up to 6 min).

Base deprotection and cleavage of oligoribonucleotides from the support were carried out by treatment with a mixture of 40% aqueous methylamine and 30% aqueous ammonia (600 µL, 1:1 v/v) in a screw-cap vial at 65 °C for 20 minutes. The resulting solution was filtered off, the support was washed with a mixture of acetonitrile, methanol and water (300 µL, 1:1:1 v/v) twice, and then combined filtrates were evaporated to dryness in a SpeedVac concentrator.

The 2'-*O*-TOM protecting groups were removed by incubation of oligoribonucleotides in a mixture of *N*-methyl-2-pyrrolidone, trimethylamine, triethylamine trihydrofluoride (200 µL, 1:1:1 v/v) at 65 °C for 2 h. The reaction was cooled in freezer briefly, and then 25 µL of 3 M sodium acetate was added followed by ~1 mL of 1-butanol. The mixture was cooled at -20 °C for 1 h. The formed precipitate of the oligonucleotide sodium salts was centrifuged (15 min, 14,000 rpm, 4 °C), washed with ethanol or acetone and air-dried.

Quality assessment of the crude RNAs was performed using anion-exchange HPLC on a Dionex DNAPac PA-100 column (4 x 250 mm); conditions: flow rate 1 mL min^{-1}; eluent A: 25 mM Tris-HCl, pH 8.0, 6 M urea, eluent B: 500 mM $NaClO_4$, 25 mM Tris-HCl, pH 8.0, 6 M urea; gradient: 0 - 60% B in 45 minutes; 60 °C, UV detection at 260 nm.

The desired oligoribonucleotides were isolated by semi-preparative anion-exchange HPLC on a Dionex DNAPac PA-100 column (9 x 250 mm); Conditions: flow rate 2 mL min^{-1}; see above. The product fractions were desalted using C18 Sep-Pak cartridges (Waters Corporation). The quality of the product was analyzed by anion-exchange HPLC and reversed-phase LC-ESI-MS. Sequences and MS data for all the obtained RNAs are shown in Supporting Table S1.

Mass spectrometry

All experiments were performed on a Finnigan LCQ Advantage MAX ion trap instrumentation connected to a Thermo Fisher Ultimate 3000 HPLC system. RNAs were analyzed in the negative-ion mode with a potential of −4 kV applied to the spray needle. LC: Sample (200 pmol RNA dissolved in 30 µL of 20 mM ethylenediamine tetraacetic acid (EDTA) solution; average injection volume: 30 µL); column (Waters XTerraMS, C18, 2.5 µm; 1.0 × 50 mm) at 21°C; flow rate: 0.1 mL/min; eluant A: 8.6 mM triethylamine (TEA), 100 mM 1,1,1,3,3,3-hexafluoroisopropanol in H_2O (pH 8.0); eluant B: methanol; gradient: 0–100% B in A within 30 min; UV detection at 254 nm.

Preparation of double labeled RNA by *N*-hydroxysuccinimide ester (NHS) chemistry and strain-promoted alkyne-azide cyloadditions (SPAAC)[1]

A solution of Sulfo-Cy5-NHS ester (26 mM, 50 µl) was prepared in anhydrous DMSO and of Sulfo-Cy3-DBCO (8.4 mM, 125 µl) in DMSO/water (50% vol/vol), respectively. 3'-end 2'-*O*-(3-azidopropyl) RNA (50 nmol) containing a 5'-(6-aminohexyl)-phosphate modification was lyophilized and dissolved in water (30µl). Then, the RNA was desalted by precipitation with sodium acetate buffer (1M, pH 5.3, 0.2 volumes of aqueous solution) and absolute ethanol (3 volumes of aqueous solution) for 2 h, at -20 °C, followed by centrifugation for 30 min at 4 °C at 12,500 RPM (Eppendorf 5430R, rotor F-45-30-11). The pellet was washed with a small amount of ethanol, centrifuged and briefly dried on high vacuum. For the labeling reaction the RNA was dissolved in labeling buffer (sodium borate buffer, 0.1 M, pH 8.5) and DMSO (50% vol/vol) to give a final concentration of 222 µM RNA and 1.6 mM Sulfo-Cy5-NHS ester (26 mM, 13.6 µl) in a total volume of 225 µl. The reaction mixture was shaken overnight in the dark. Subsequently, the RNA was precipitated by the addition of sodium acetate buffer (1 M, pH 5.3, 0.2 volumes of labeling reaction) and ethanol (2.5 volumes of labeling reaction), for 2h at -20 °C. The excess of unreacted hydrolyzed dye was removed by centrifugation for 30 min at 4 °C at 12,500 RPM. For the second labeling step, the single labeled RNA was dissolved in DMSO/water (50% vol/vol) to give a total concentration of 333 µm RNA and 667 µM Sulfo-Cy-3 DBCO (8.4 mM, 14.9 µl) in a final volume of 150 µl. The reaction mixture was shaken for 3 h at room temperature in the dark.

Monitoring and purification of the reactions was performed by anion exchange HPLC. In case of incomplete reactions, the reaction mixture was precipitated and the corresponding labeling reaction was repeated without previous HPLC purification steps.

Kinetics of ribozyme cleavage (HPLC assay)[2,3]

Nanomole aliquots (2.64 nmol) of the ribozyme and substrate strand were taken from aqueous stock solutions, mixed and lyophilized. The RNA was dissolved in 33.6 µl (78.6 µM) nanopure water, heated to 90 °C for 2 min and allowed to cool to room temperature. For time point 0 (prior to the Mg^{2+} induced cleavage reaction) 2.8 µl (220 pmol) of the RNA solution were diluted with nanopure water to a total volume of 100 µl. Further, 6.6 µl HEPES buffer (200mM, pH 7.5) and 2.2 µl KCl solution (2M) were added to the RNA solution. Subsequently, the cleavage reaction was initiated by the addition of 4.4 µl $MgCl_2$ solution (20 mM), leading to total concentrations of 55.0 µM RNA, 30 mM HEPES, 100mM KCl and 2 mM Mg^{2+} in a total volume of 44.0 µl. Samples (4 µL) were drawn after the indicated time points, quenched with EDTA solution (4 µl, 40 mM) and diluted with water to a final volume of 100 µl.

Kinetics of ribozyme cleavage (FRET assay)

All measurements were performed on a Cary Eclipse spectrometer (Varian, Australia) equipped with a peltier block and a magnetic stirring device.

Equivalent amounts (60 pmol) of Cy5/Cy3 labeled substrate strand and ribozyme strand were lyophilized. The RNA was dissolved in 120 µl MOPS buffer (50 mM KMOPS pH 7.5, 100 mM KCl) to reach a final concentration of 0.5 µM, heated to 90 °C and allowed to cool to room temperature for 10 minutes. The solution was transferred into a quartz cuvette. Subsequently, the fluorescence trace was recorded by using following parameters: excitation: 548 nm, emission: 662 nm data point collection: 0.2 s, slit width 10 nm, detector voltage: 690, temperature: 20 °C. After 1 min of base line detection, the cleavage reaction was manually initiated by the addition of $MgCl_2$ solution (1.2 µl, 1M) to gain a final concentration of 10 mM Mg^{2+}. The decreasing FRET intensity was monitored until a constant fluorescence value was achieved as a result of total cleavage of the substrate strand. Three independent measurements were performed for all ribozyme variants of interest. The fluorescence data were fitted by a three-parameter (A_1, A_2 and k_{obs}) single-exponential equation (1) with A_1 as final fluorescence, and $A_2 \cdot exp(-k_{obs} \cdot t)$ as change in fluorescence over time (t) at the observed rate k_{obs}. Data processing was performed by using the software package OriginPro 2018 (OriginLab, USA).

$$F(t) = A_1 + A_2 \cdot e^{(k_{obs} \cdot t)} \qquad (1)$$

Determination of pH-rate profiles for ribozyme cleavage (FRET assay/HPLC assay)[4,5,6]

The experimental setup was performed as described above (*Kinetics of ribozyme cleavage (FRET assay and HPLC assay, respectively)*). The pH series for the ribozyme variants were measured in corresponding buffer systems (Supporting Table S1), with three independent measurements for each pH value. The according pH values were fitted against the observed rate constants (k_{obs}) by using a three-parametric (k_{max}, pK_a^A, pK_a^B) equation (2), representing a bell-shaped pH profile, revealing a maximum at ($pK_a^A + pK_a^B$)/2. K_{max} reflects the maximum of the cleavage activity in the case of complete protonation of the general acid and complete deprotonation of the general base, independent of the pH value. Consequently, pK_a^A denotes the pK_a of the acidic group that is deprotonated and pK_a^B denotes the pK_a of the basic unit that is protonated, respectively. In the presence of an influencer a cubic cooperative model, reflected by equation (3) was applied, including a p*K*a of the influencer species termed as pK_a^I, leading to pK_a shifts denoted as ΔpK_a^{coop} and two plateaus for the cleavage activity that refer

to k_1 and k_2. As a consequence of the small difference among the apparent pK_a values (ΔpK_a~1) the rate-pH profiles remain sharp, limiting the accuracy of the predicted values and errors, respectively. Therefore, we fixed k_{max} and k1, respectively, to a certain value, that showed the most precise fit for our data points with reliable errors. Data processing was performed by using the software package OriginPro 2018 (OriginLab, USA).

$$k_{obs} = \frac{k_{max}}{\left(1 + 10^{(pK_a^B - pH)}\right) + 10^{(pK_a^B - pK_a^A)} + 10^{(pH - pK_a^A)}}$$

(2)

$$k_{obs} = (k_1 + k_2 * 10^{((pK_a^I + \Delta pK_a^{coop}) - pH)}) \,/\, [(1 + 10^{(pK_a^B - pH)} + 10^{(pK_a^B - pK_a^A)} + 10^{(pH - pK_a^A)} + 10^{((pK_a^I + \Delta pK_a^{coop}) - pH)} + 10^{((pK_a^B - \Delta pK_a^{coop}) + (pK_a^I + \Delta pK_a^{coop}) - 2*pH)} + 10^{((pK_a^B - \Delta pK_a^{coop}) + (pK_a^I + \Delta pK_a^{coop}) - pK_a^A - pH))} + 10^{((pK_a^I + \Delta pK_a^{coop}) - pK_a^A)})\,]$$

(3)

Supporting Figures

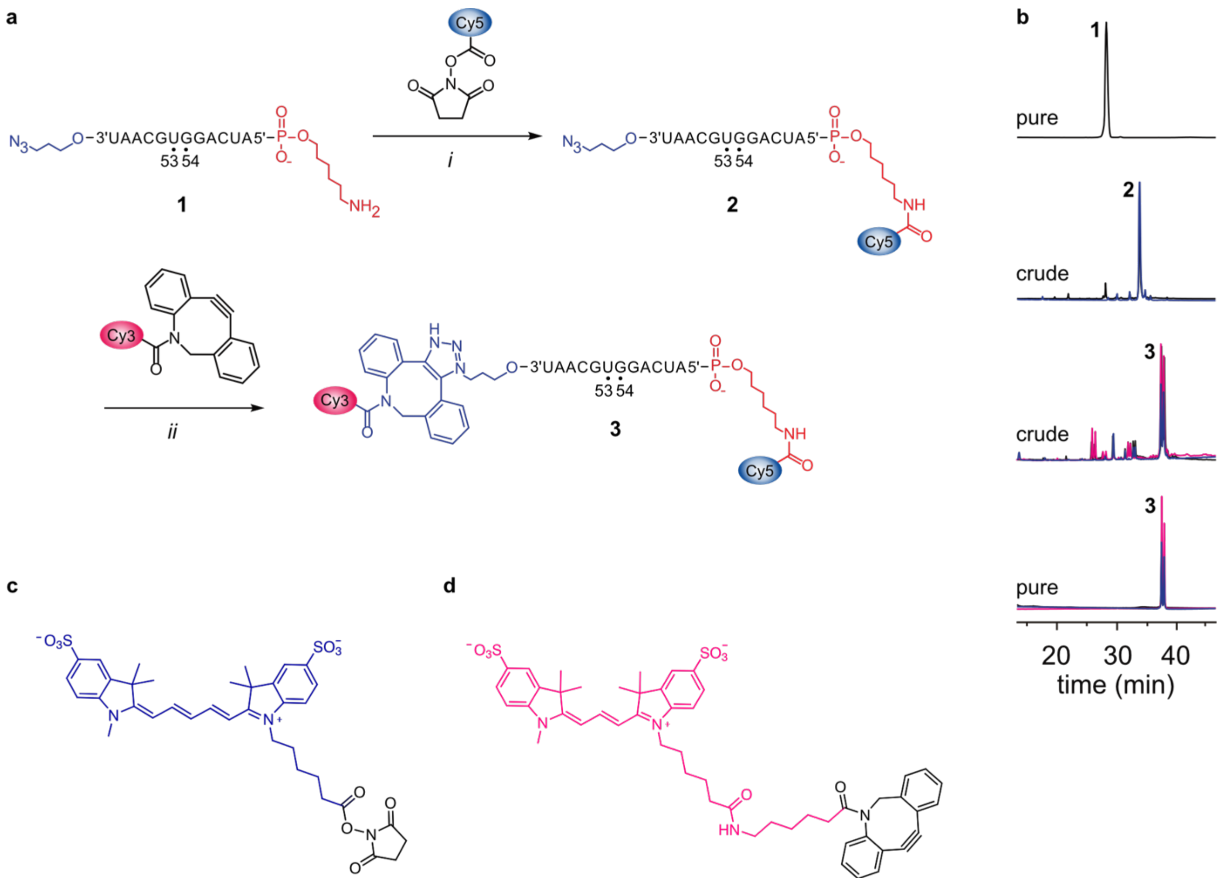

Supporting Figure S1. **a**) Concept for double labeling of 3'-terminal 2'-*O*-(2-azidoethyl) and 5'-terminal 5'-*O*-*PO*$_3$-(6-aminohexyl) modified *env25* pistol ribozyme substrate strand. *i*) N-hydroxysuccinimide (NHS) ester based Cy5 conjugation: conditions: 0.1 M $NaBO_3$ buffer pH 8.5/DMSO (1:1), r.t., overnight, in the dark; *ii*) strain-promoted alkyne azide conjugation (SPAAC) of Cy3: conditions: H_2O/DMSO, r.t., 3 h, in the dark; Note: For the non-cleavable substrate strand a 3'-terminal 2'-O-(2-azidoethyl) and 5'-terminal 5'-O-PO_3-(6-aminohexyl) modified G53dG mutant was prepared and labeled equally; **b**) HPLC profiles of the pure unlabeled RNA **1,** the crude Cy5 labeled RNA **2** (UV traces: 260 nm (black) & 646 nm (blue), followed by the crude reaction mixture after strain-promoted alkyne azide conjugation (SPAAC) of Cy3 (UV traces: 260 nm (black), 646 nm (blue) & 548 nm (magenta), purified double labeled RNA **3** (UV traces: 260 nm (black), 646 nm (blue) & 548 nm (magenta); **c**) Sulfo-cyanine-5-NHS-ester, MW=738.85 g/mol, absorption maximum= 646 nm, emission maximum: 662nm, ε=271000 L^{-1} mol cm^{-1}; **d**) Sulfo-cyanine-3-DBCO, MW=916.14 g/mol, absorption maximum: 548 nm, emission maximum 563 nm, ε=162000 L^{-1} mol cm^{-1}

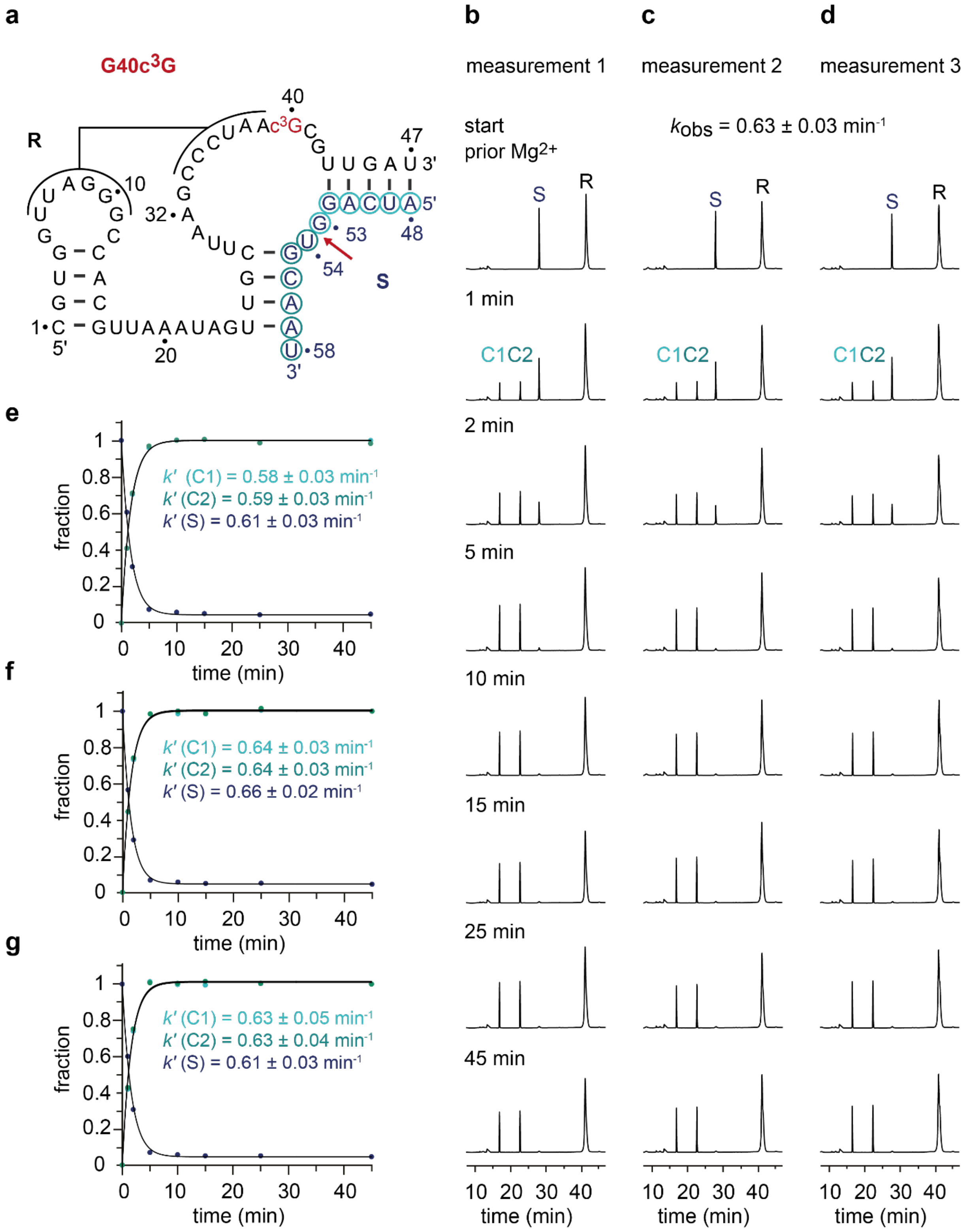

Supporting Figure S2. Self-cleavage of G40c^{3}G *env25* pistol ribozyme mutant. **a**) G40c^{3}G modified RNA set-up; **b-d**) Anion exchange HPLC profiles of the reaction time course of three independent measurements; reaction conditions: c(RNA) = 55 µM each RNA strand (1:1 ratio); 2 mM $MgCl_2$, 100 mM KCl, 30 mM HEPES, pH 7.5, 25 °C. The reaction was stopped at the indicated time points by drawing a 4 µL sample and mixing it with 4 µL of stop solution (40 mM Na_2EDTA), followed by dilution with 100 µL of water. HPLC conditions: Dionex DNAPac column (4x250 mm), 60 °C, 1 ml min^{-1}, 0–60% buffer B in 45 min. Buffer A: Tris–HCl (25 mM), urea (6 M), pH 8.0. Buffer B: Tris–HCl (25 mM), urea (6 M), $NaClO_4$ (0.5 M), pH 8.0. **e-g**) Estimation of observed rates from fractions (S, C1, and C2) obtained by HPLC analysis.

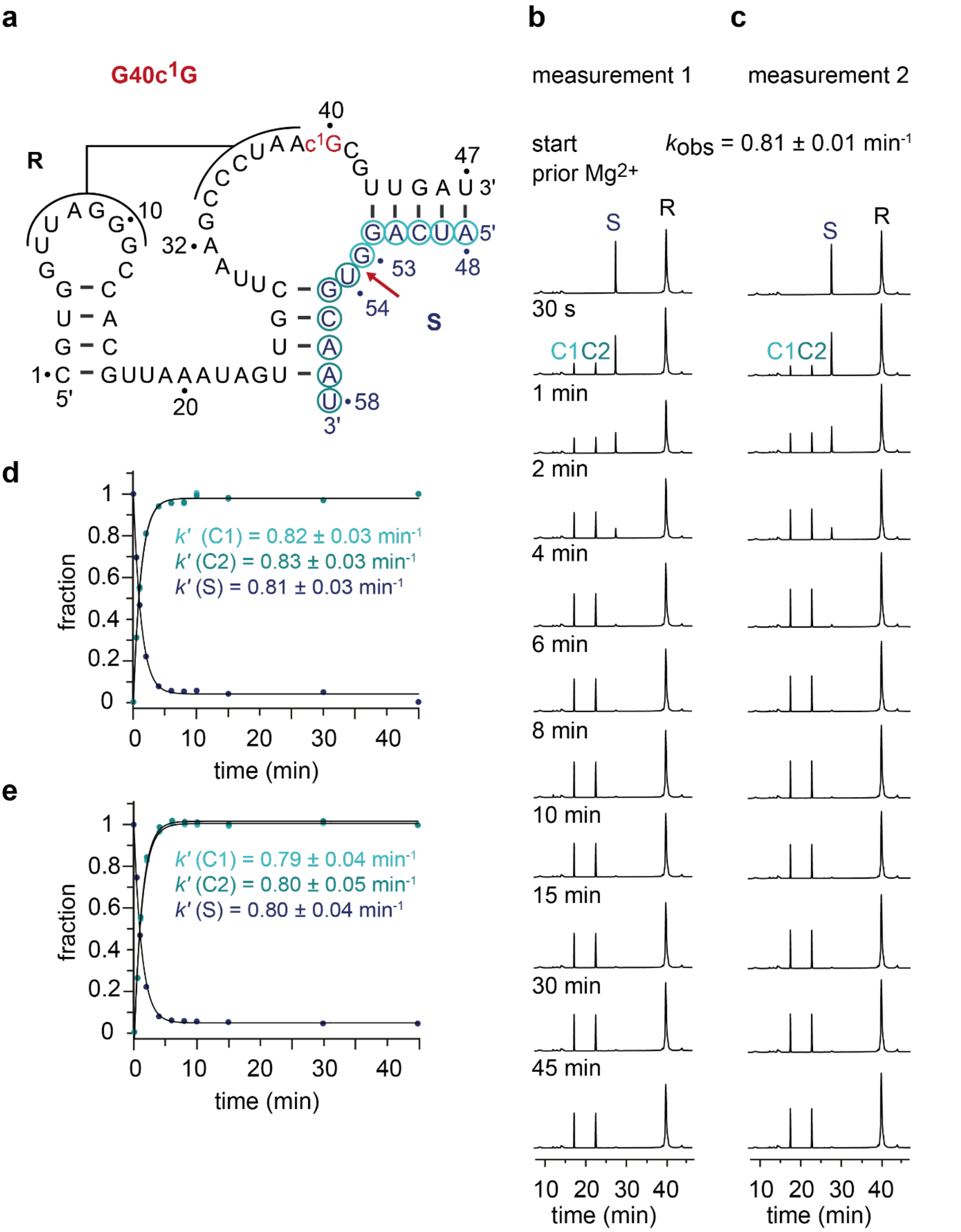

Supporting Figure S3. Self-cleavage of G40c^{1}G *env25* pistol ribozyme mutant. **a**) G40c^{1}G modified RNA set-up; **b-c**) Anion exchange HPLC profiles of the reaction time course of two independent measurements; reaction conditions: c(RNA) = 55 µM each RNA strand (1:1 ratio); 2 mM $MgCl_2$, 100 mM KCl, 30 mM HEPES, pH 7.5, 25 °C. The reaction was stopped at the indicated time points by drawing a 4 µL sample and mixing it with 4 µL of stop solution (40 mM Na_2EDTA), followed by dilution into 100 µL of water. HPLC conditions: Dionex DNAPac column (4x250 mm), 60 °C, 1 ml min^{-1}, 0–60% buffer B in 45 min. Buffer A: Tris–HCl (25 mM), urea (6 M), pH 8.0. Buffer B: Tris–HCl (25 mM), urea (6 M), $NaClO_4$ (0.5 M), pH 8.0. **d-e**) Estimation of observed rates from fractions (S, C1, and C2) obtained by HPLC analysis.

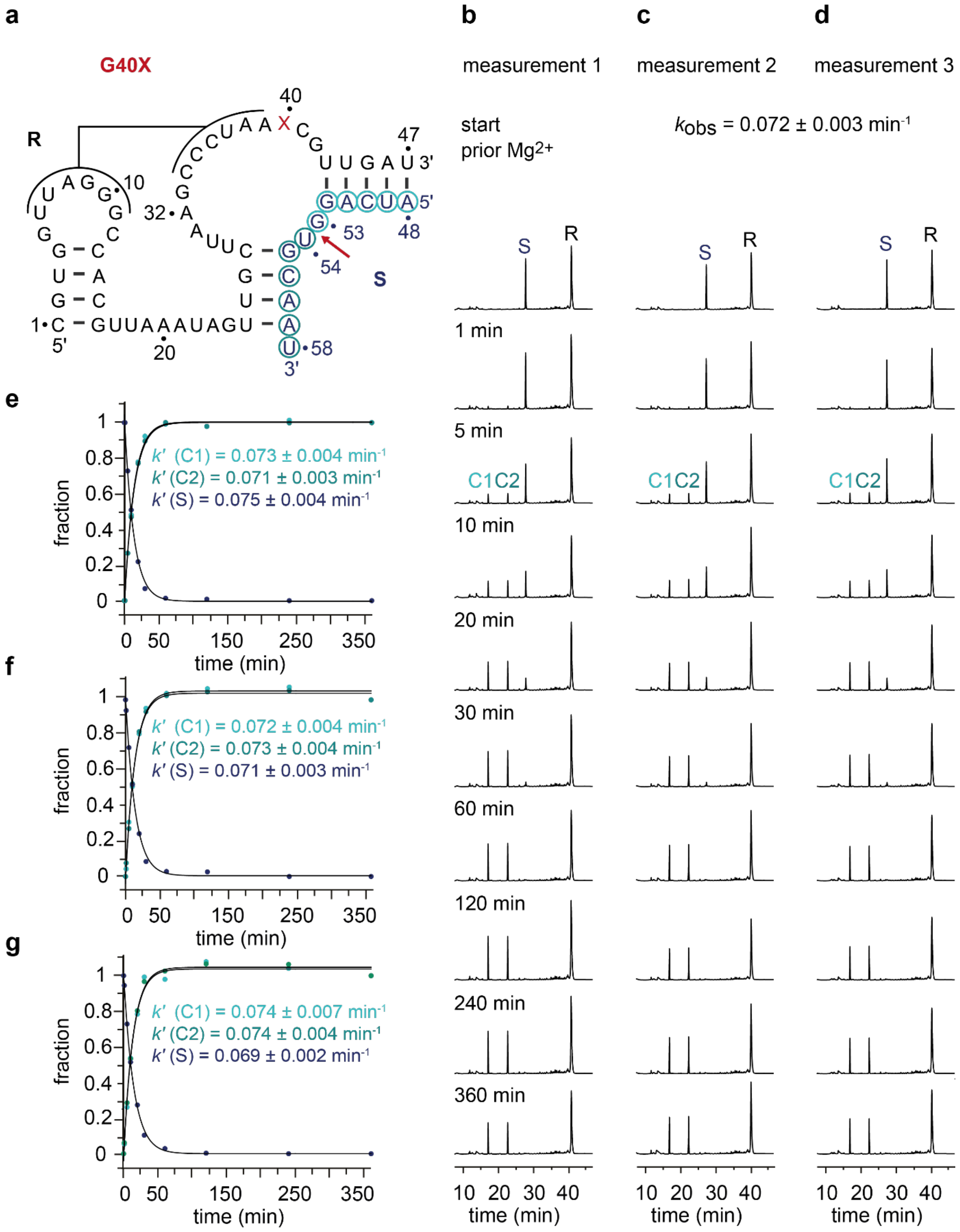

Supporting Figure S4. Self-cleavage of G40Xn *env25* pistol ribozyme mutant. **a**) G40Xn modified RNA set-up; **b-c**) Anion exchange HPLC traces of the reaction time course of three independent measurements; reaction conditions: c(RNA) = 55 µM each RNA strand (1:1 ratio); 2 mM $MgCl_2$, 100 mM KCl, 30 mM HEPES, pH 7.5, 25 °C. The reaction was stopped at the indicated time points by drawing a 4 µL sample and mixing it with 4 µL of stop solution (40 mM Na_2EDTA), followed by dilution with 100 µL of water. HPLC conditions: Dionex DNAPac column (4x250 mm), 60 °C, 1 ml min^{-1}, 0–60% buffer B in 45 min. Buffer A: Tris–HCl (25 mM), urea (6 M), pH 8.0. Buffer B: Tris–HCl (25 mM), urea (6 M), $NaClO_4$ (0.5 M), pH 8.0. **d-e**) Estimation of observed rates from fractions (S, C1, and C2) obtained by HPLC analysis.

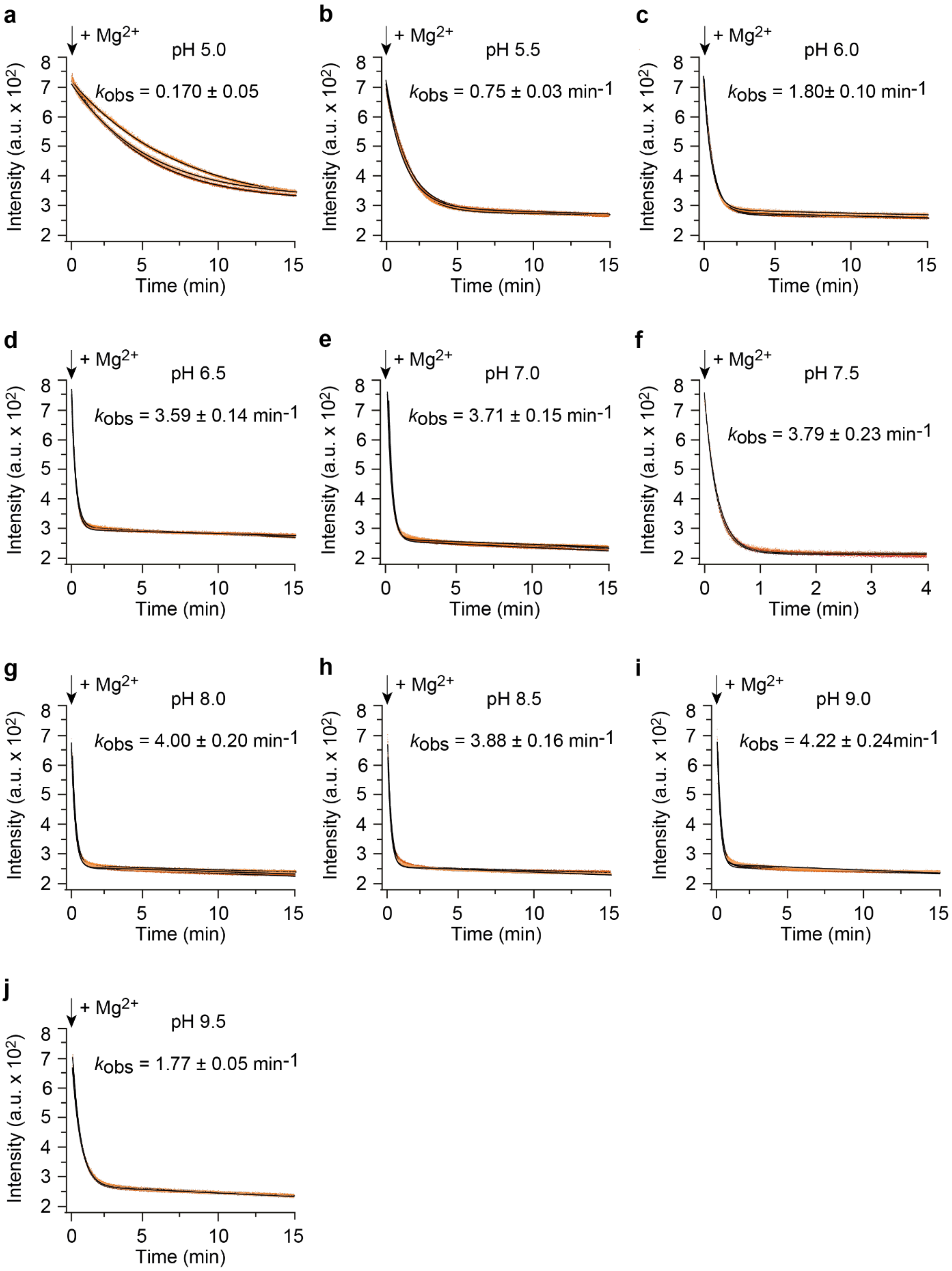

Supporting Figure S5. Fluorescence emission response of acceptor dye upon Mg^{2+} induced self-cleavage of wildtype *env25* pistol ribozyme mutant. **a-j**) Time course of fluorescence response at different pH values ranging from pH 5 to pH 9.5 leading to according rate constants (the values given represent the mean of three independent measurements for each pH value); conditions: c(RNA) = 0.5 µM of each RNA strand (1:1 ratio); 10 mM MgCl2, 25 °C, pH 5 - 6.5: 100 mM KCl, 50 mM MES; pH7 - 7.5: 100 mM KCl; 50 mM KMOPS; pH 8 - 8.5: 100 mM KCl, 50 mM TRIS; pH 9 - 9.5: 100 mM KCl, 50 mM TAPS.

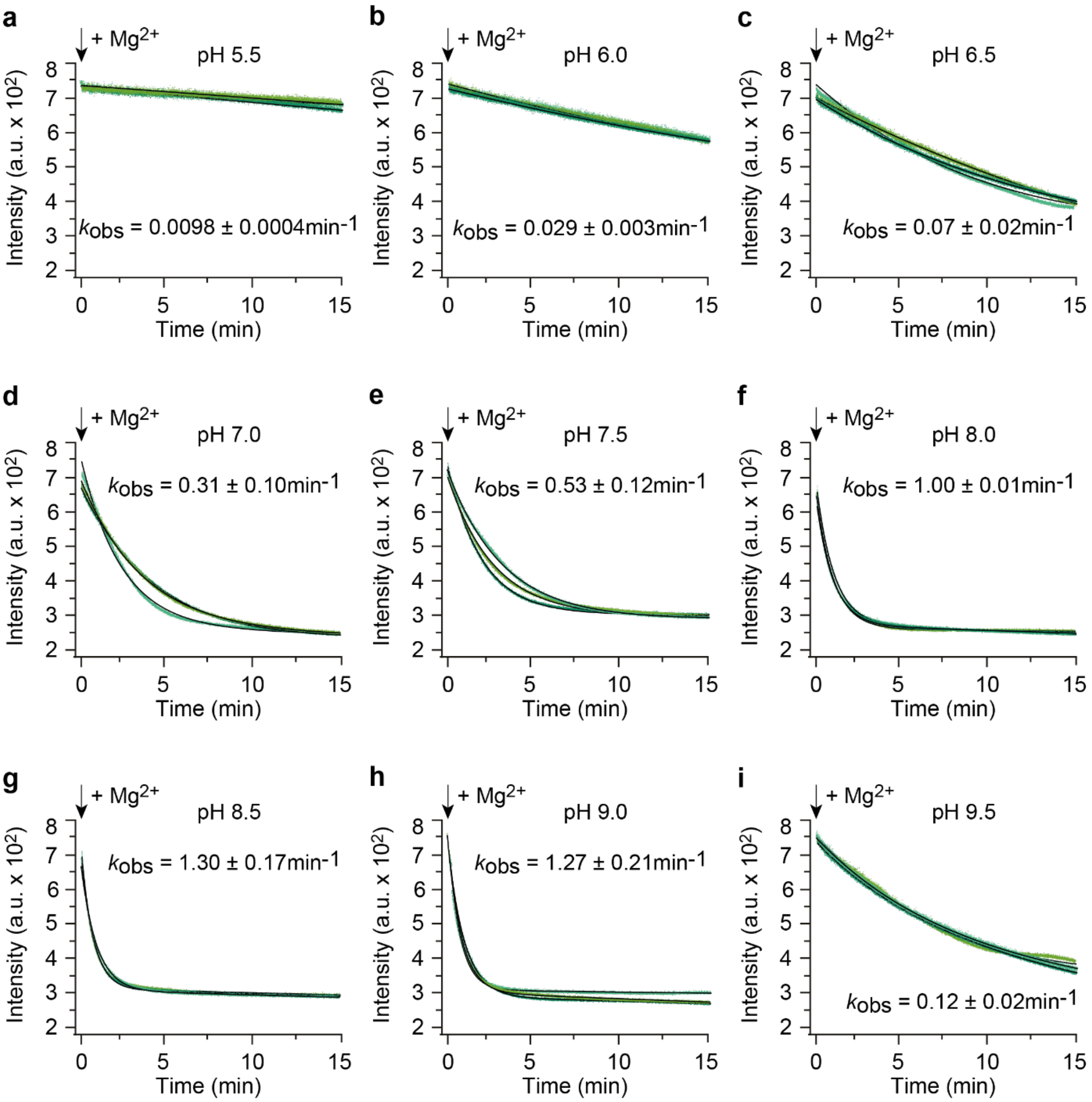

Supporting Figure S6. Fluorescence emission response of acceptor dye upon Mg^{2+} induced self-cleavage of G40c^{3}G *env25* pistol ribozyme mutant. a-i) Time course of fluorescence response at different pH values ranging from pH 5.5 to pH 9.5 leading to according rate constants (the values given are the mean of three independent measurements for each pH value); conditions: c(RNA) = 0.5 µM of each RNA strand (1:1 ratio); 10 mM MgCl2, 25 °C, pH 5.5 - 6.5: 100 mM KCl, 50 mM MES; pH7 - 7.5: 100 mM KCl; 50 mM KMOPS; pH 8 - 8.5: 100 mM KCl, 50 mM TRIS; pH 9 - 9.5: 100 mM KCl, 50 mM TAPS.

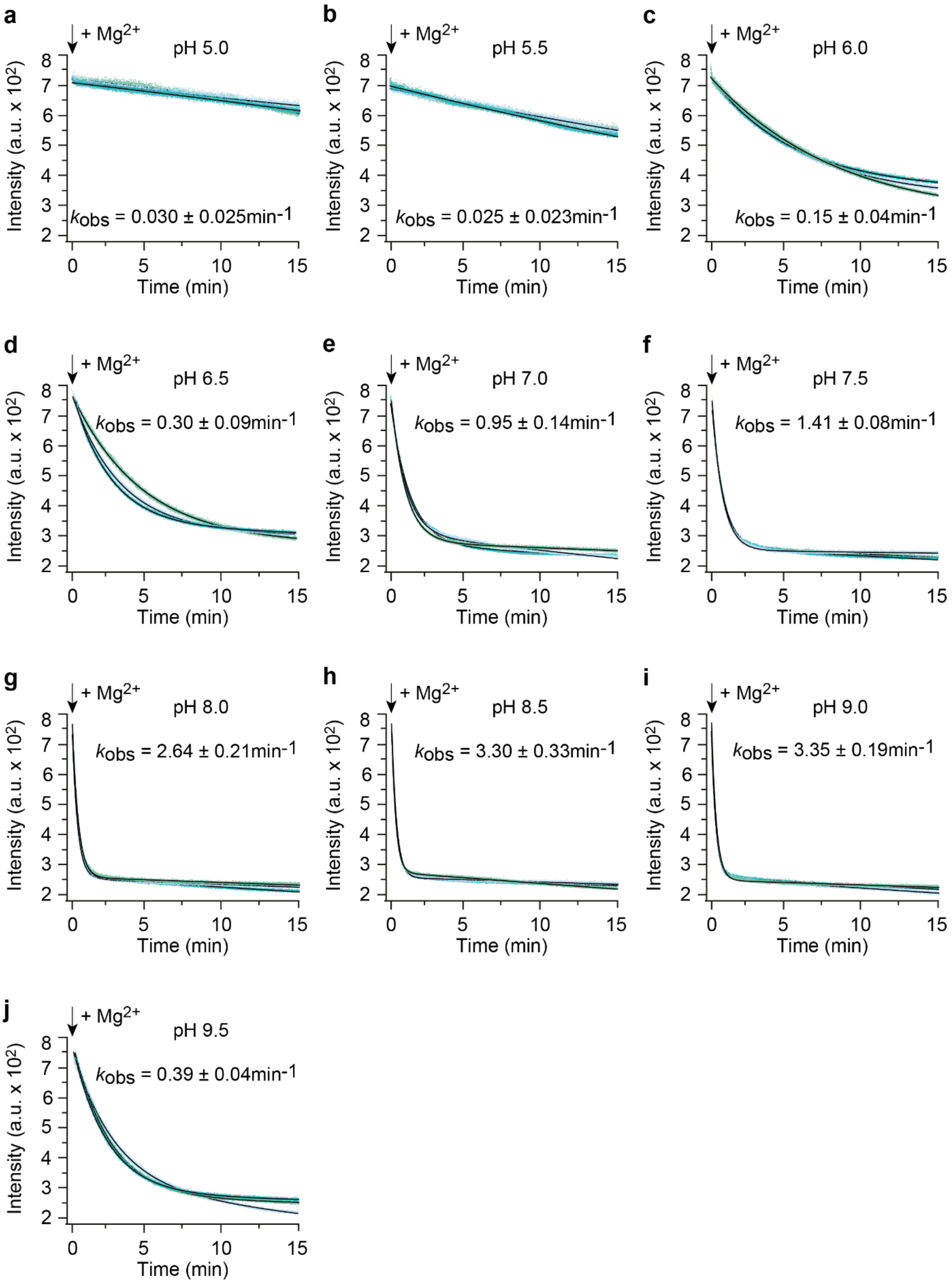

Supporting Figure S7. Fluorescence emission response of acceptor dye upon Mg^{2+} induced self-cleavage of G40c^{1}G *env25* pistol ribozyme mutant. a-j) Time course of fluorescence response at different pH values ranging from pH 5 to pH 9.5 leading to according rate constants (the values given are the mean of three independent measurements for each pH value); conditions: c(RNA) = 0.5 µM of each RNA strand (1:1 ratio); 10 mM MgCl2, 25 °C, pH 5 - 6.5: 100 mM KCl, 50 mM MES; pH7 - 7.5: 100 mM KCl; 50 mM KMOPS; pH 8 - 8.5: 100 mM KCl, 50 mM TRIS; pH 9 - 9.5: 100 mM KCl, 50 mM TAPS.

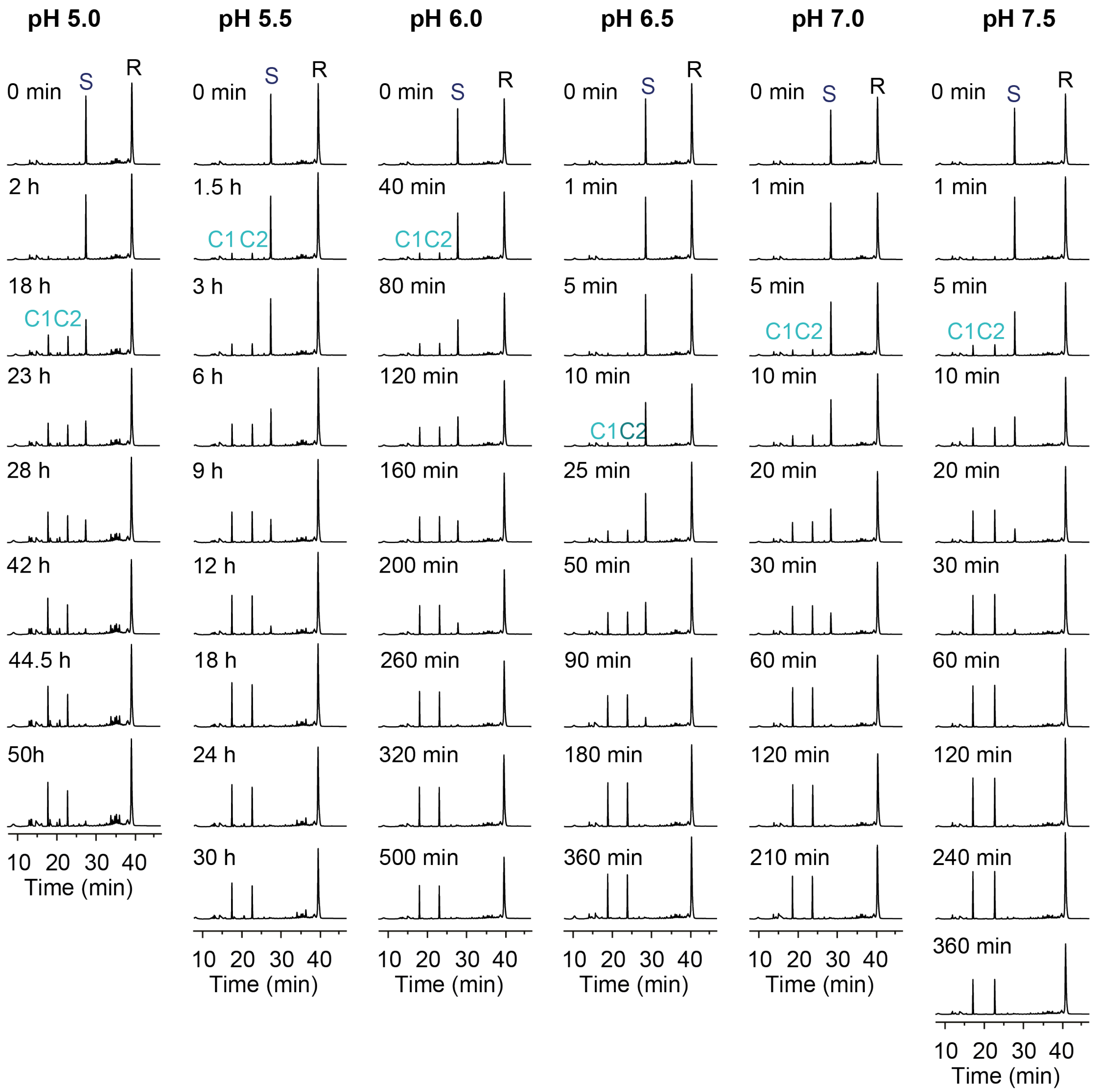

Supporting Figure S8. Self-cleavage of G40X *env25* pistol ribozyme mutant at according pH values. Anion exchange HPLC traces of the reaction time course.; reaction conditions: c(RNA) = 55 µM each RNA strand (1:1 ratio); 2 mM $MgCl_2$, 25 °C; pH 5 - 6.5: 100 mM KCl, 30 mM MES, pH 7 - 7.5: 100 mM KCl, 30 mM MOPS, 25 °C. The reaction was stopped at the indicated time points by drawing a 4 µL sample and mixing it with 4 µL of stop solution (40 mM Na_2EDTA), followed by dilution with 100 µL of water. HPLC conditions: Dionex DNAPac column (4x250 mm), 60 °C, 1 ml min^{-1}, 0–60% buffer B in 45 min. Buffer A: Tris–HCl (25 mM), urea (6 M), pH 8.0. Buffer B: Tris–HCl (25 mM), urea (6 M), $NaClO_4$ (0.5 M), pH 8.0.

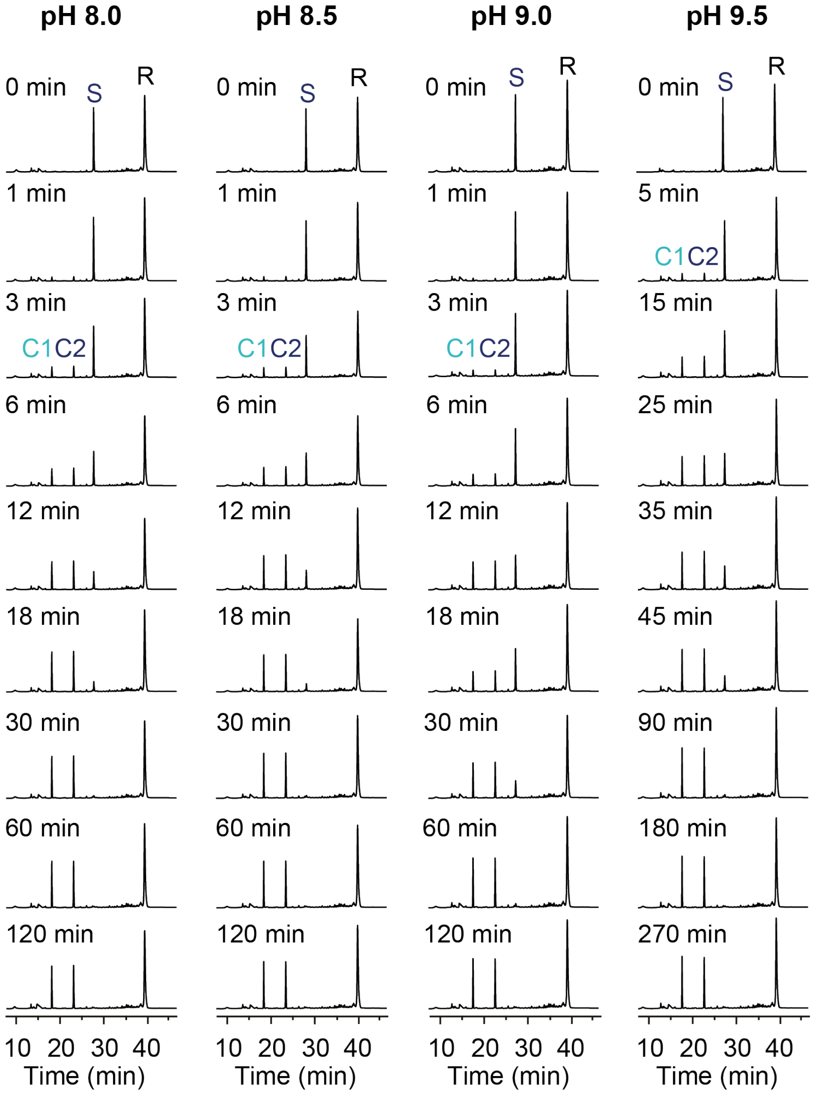

Supporting Figure S9. Self-cleavage of G40X *env25* pistol ribozyme mutant at according pH values. Anion exchange HPLC traces of the reaction time course.; reaction conditions: c(RNA) = 55 µM each RNA strand (1:1 ratio); 2 mM $MgCl_2$, 25 °C; pH 8 - 8.5: 100 mM KCl, 30 mM TRIS, pH 9 - 9.5: 100 mM KCl, 30 mM TAPS, 25 °C. The reaction was stopped at the indicated time points by drawing a 4 µL sample and mixing it with 4 µL of stop solution (40 mM Na_2EDTA), followed by dilution with 100 µL of water. HPLC conditions: Dionex DNAPac column (4x250 mm), 60 °C, 1 ml min^{-1}, 0–60% buffer B in 45 min. Buffer A: Tris–HCl (25 mM), urea (6 M), pH 8.0. Buffer B: Tris–HCl (25 mM), urea (6 M), $NaClO_4$ (0.5 M), pH 8.0.

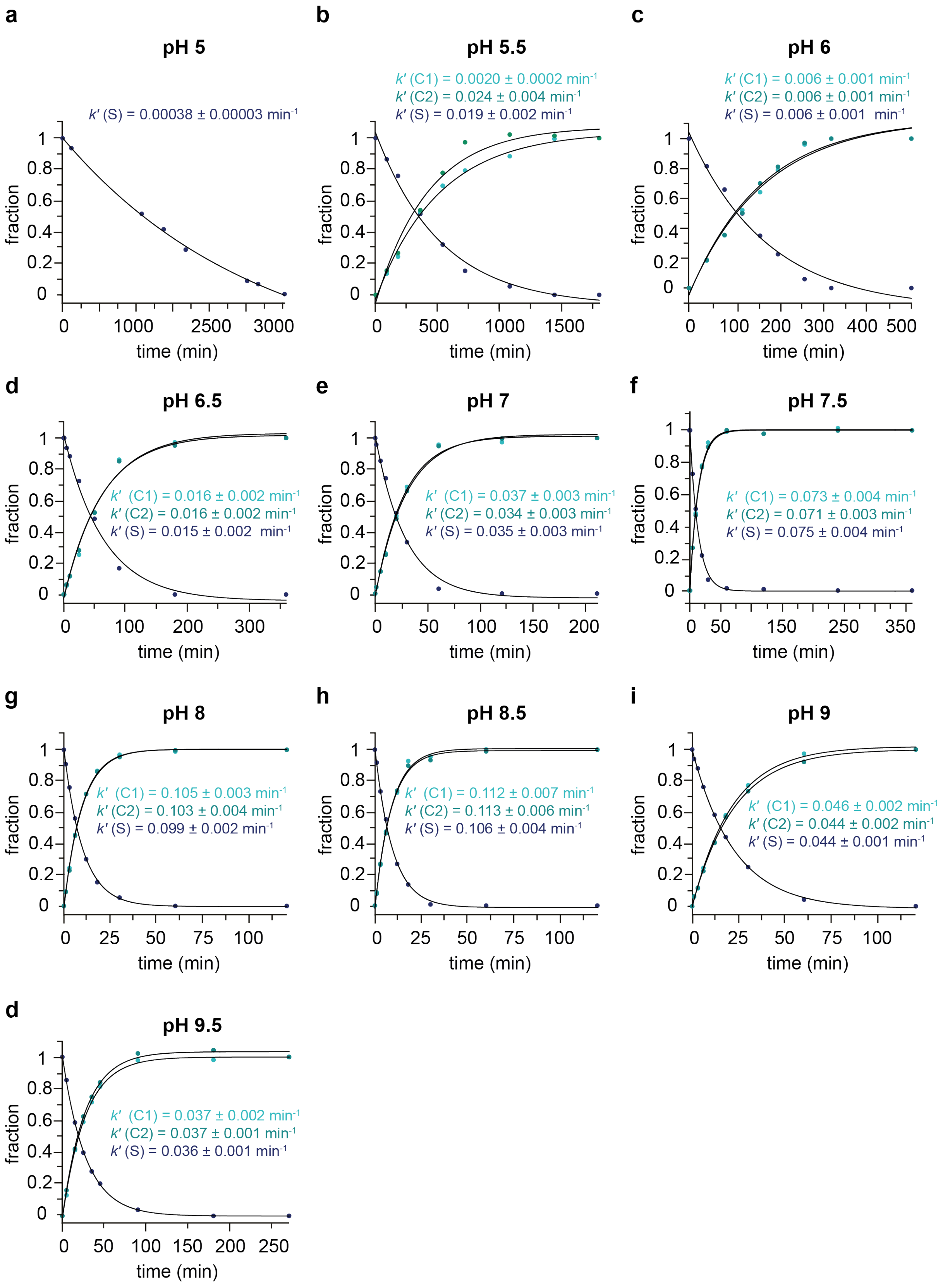

Supporting Figure S10. Determination of the pH dependent cleavage rates (pH 5 – 9.5) for the self-cleavage of the G40X *env25* pistol ribozyme mutant. **a-d**) Estimation of the observed rate over the indicated pH range from the fractions (S, C1, and C2) obtained by HPLC analysis.

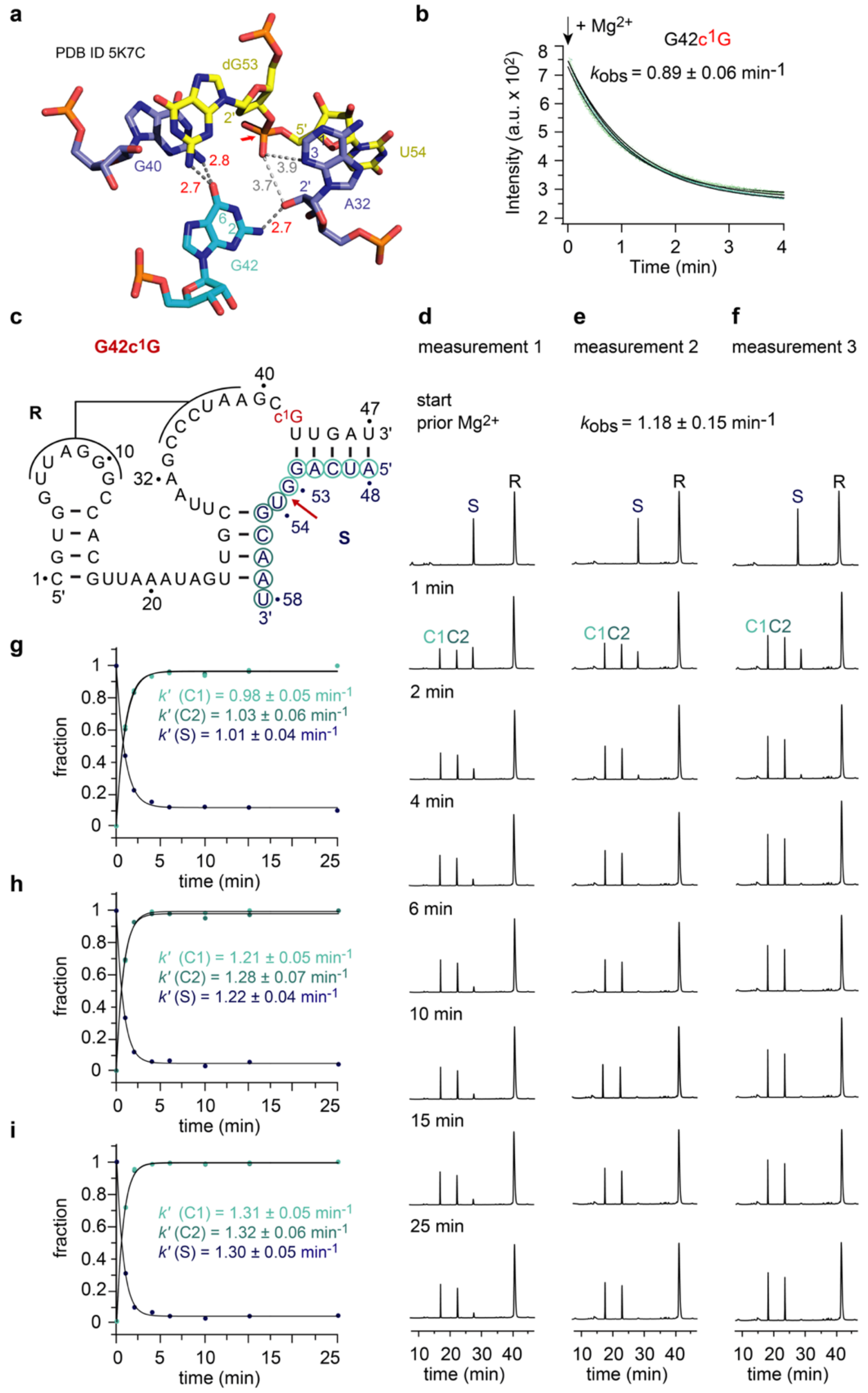

Supporting Figure S11. Self-cleavage of G42c[1]G *env25* pistol ribozyme mutant. **a**) Three-dimensional structure of the active site with emphasis on G42 bridging G40 and A32 (PDB ID 5K7C). Fluorescence emission response of acceptor dye upon Mg^{2+} induced self-cleavage of G42c[1]G mutant; conditions: c(RNA) = 0.5 µM of each RNA strand (1:1 ratio); 10 mM $MgCl_2$, 25 °C, pH 7.5: 100 mM KCl; **b**) G42c[1]G RNA set-up for HPLC cleavage assay; **c-e**) Anion exchange HPLC profiles of the reaction time course of three independent measurements; reaction conditions: c(RNA) = 55 µM each RNA strand (1:1 ratio); 2 mM $MgCl_2$, 100 mM KCl, 30 mM HEPES, pH 7.5, 25 °C. The reaction was stopped at the indicated time points by drawing a 4 µL sample and mixing it with 4 µL of stop solution (40 mM Na_2EDTA), followed by dilution with 100 µL of water. HPLC conditions: Dionex DNAPac column (4x250 mm), 60 °C, 1 ml min^{-1}, 0–60% buffer B in 45 min. Buffer A: Tris–HCl (25 mM), urea (6 M), pH 8.0. Buffer B: Tris–HCl (25 mM), urea (6 M), $NaClO_4$ (0.5 M), pH 8.0. **f-h**) Estimation of observed rates from fractions (S, C1, and C2) obtained by HPLC analysis.

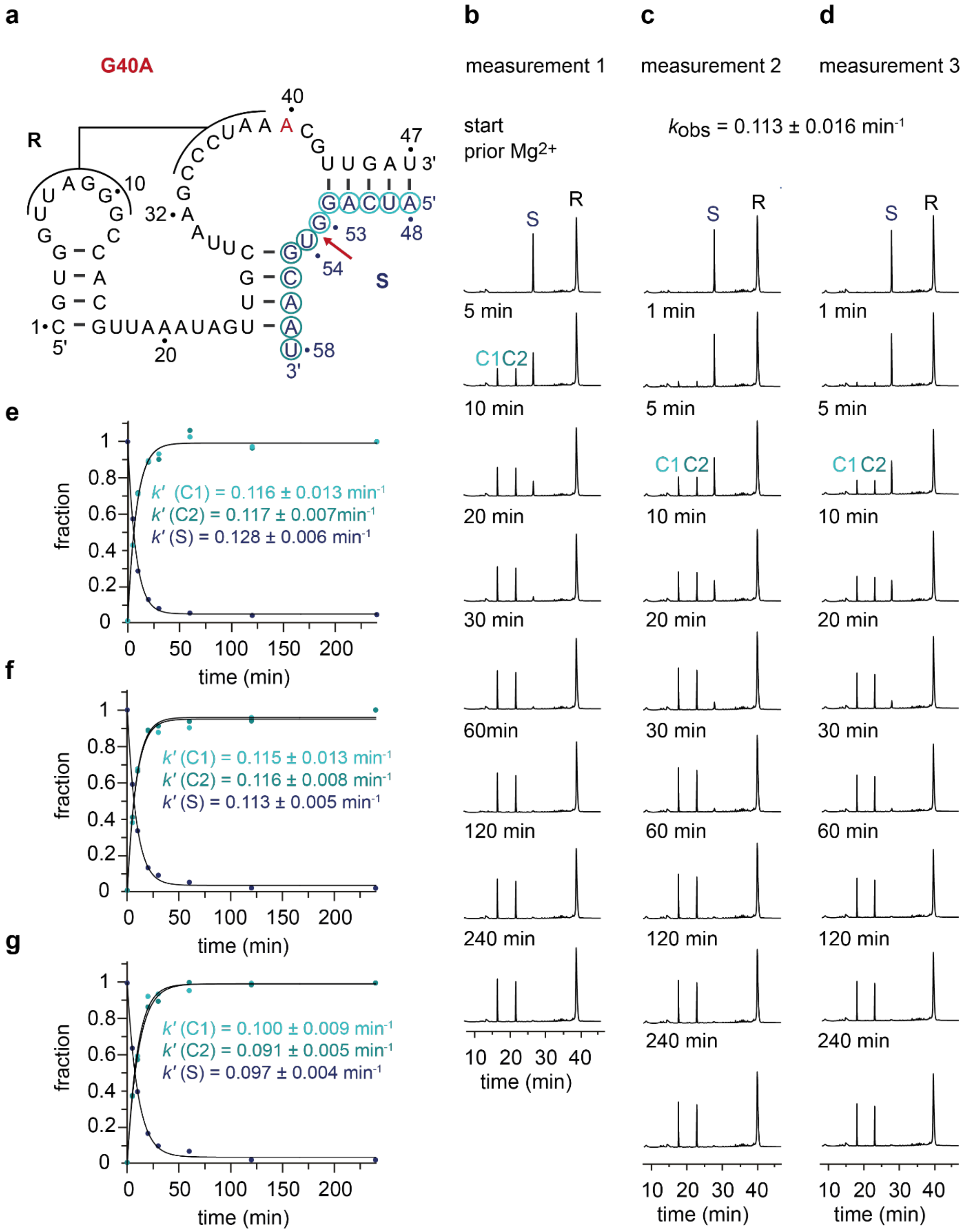

Supporting Figure S12. Self-cleavage of G40A *env25* pistol ribozyme mutant. **a**) G40A modified RNA set-up; **b-d**) Anion exchange HPLC profiles of the reaction time course of three independent measurements; reaction conditions: c(RNA) = 55 µM each RNA strand (1:1 ratio); 2 mM $MgCl_2$, 100 mM KCl, 30 mM HEPES, pH 7.5, 25 °C. The reaction was stopped at the indicated time points by drawing a 4 µL sample and mixing it with 4 µL of stop solution (40 mM Na_2EDTA), followed by dilution into 100 µL of water. HPLC conditions: Dionex DNAPac column (4x250 mm), 60 °C, 1 ml min^{-1}, 0–60% buffer B in 45 min. Buffer A: Tris–HCl (25 mM), urea (6 M), pH 8.0. Buffer B: Tris–HCl (25 mM), urea (6 M), $NaClO_4$ (0.5 M), pH 8.0. **d-e**) Estimation of observed rates from fractions (S, C1, and C2) obtained by HPLC analysis.

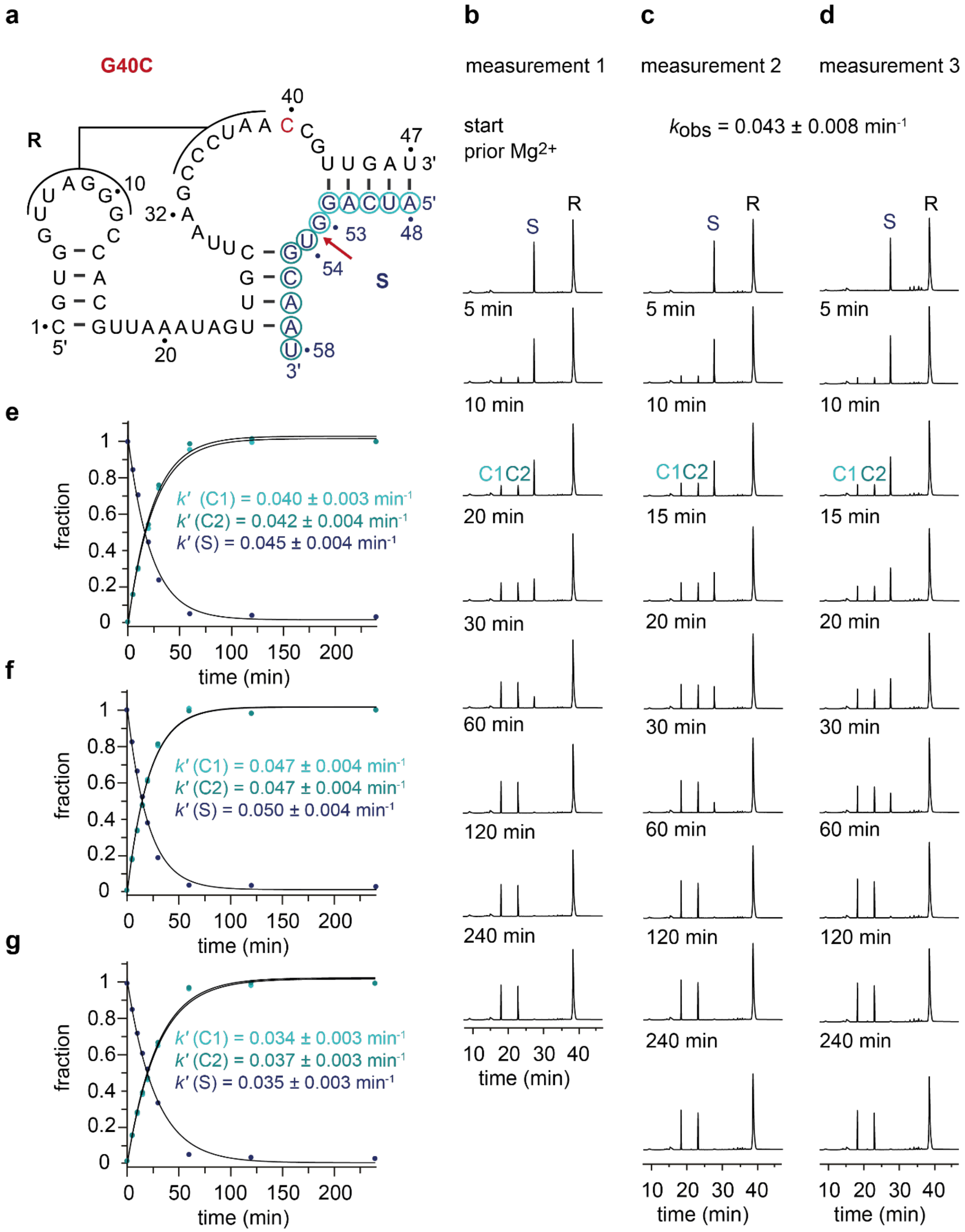

Supporting Figure S13. Self-cleavage of G40C *env25* pistol ribozyme mutant. **a**) G40C modified RNA set-up; **b-d**) Anion exchange HPLC profiles of the reaction time course of three independent measurements; reaction conditions: c(RNA) = 55 µM each RNA strand (1:1 ratio); 2 mM $MgCl_2$, 100 mM KCl, 30 mM HEPES, pH 7.5, 25 °C. The reaction was stopped at the indicated time points by drawing a 4 µL sample and mixing it with 4 µL of stop solution (40 mM Na_2EDTA), followed by dilution into 100 µL of water. HPLC conditions: Dionex DNAPac column (4x250 mm), 60 °C, 1 ml min^{-1}, 0–60% buffer B in 45 min. Buffer A: Tris–HCl (25 mM), urea (6 M), pH 8.0. Buffer B: Tris–HCl (25 mM), urea (6 M), $NaClO_4$ (0.5 M), pH 8.0. **d-e**) Estimation of observed rates from fractions (S, C1, and C2) obtained by HPLC analysis.

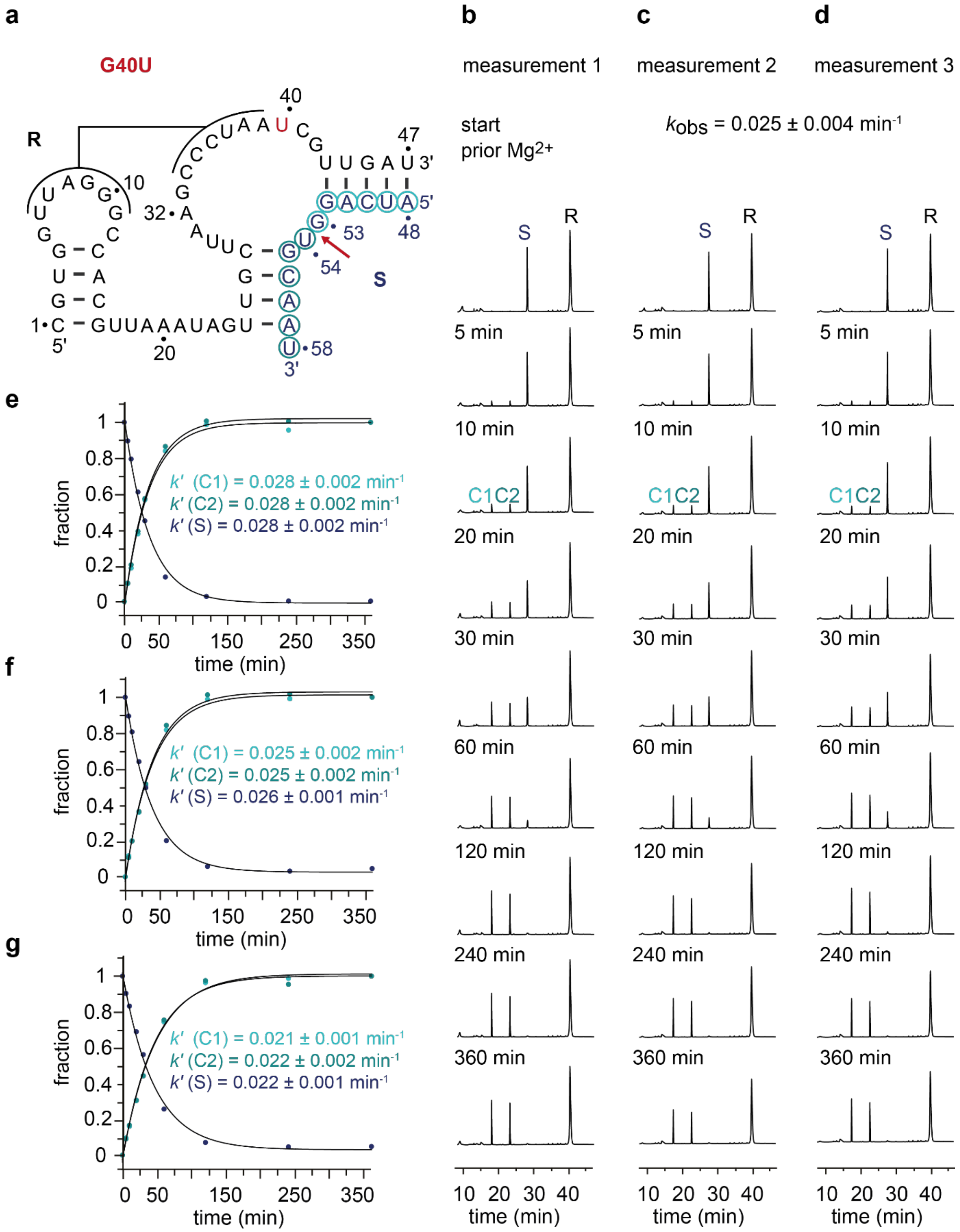

Supporting Figure S14. Self-cleavage of G40U *env25* pistol ribozyme mutant. **a**) G40U modified RNA set-up; **b-d**) Anion exchange HPLC profiles of the reaction time course of three independent measurements; reaction conditions: c(RNA) = 55 µM each RNA strand (1:1 ratio); 2 mM $MgCl_2$, 100 mM KCl, 30 mM HEPES, pH 7.5, 25 °C. The reaction was stopped at the indicated time points by drawing a 4 µL sample and mixing it with 4 µL of stop solution (40 mM Na_2EDTA), followed by dilution into 100 µL of water. HPLC conditions: Dionex DNAPac column (4x250 mm), 60 °C, 1 ml min^{-1}, 0–60% buffer B in 45 min. Buffer A: Tris–HCl (25 mM), urea (6 M), pH 8.0. Buffer B: Tris–HCl (25 mM), urea (6 M), $NaClO_4$ (0.5 M), pH 8.0. **d-e**) Estimation of observed rates from fractions (S, C1, and C2) obtained by HPLC analysis.

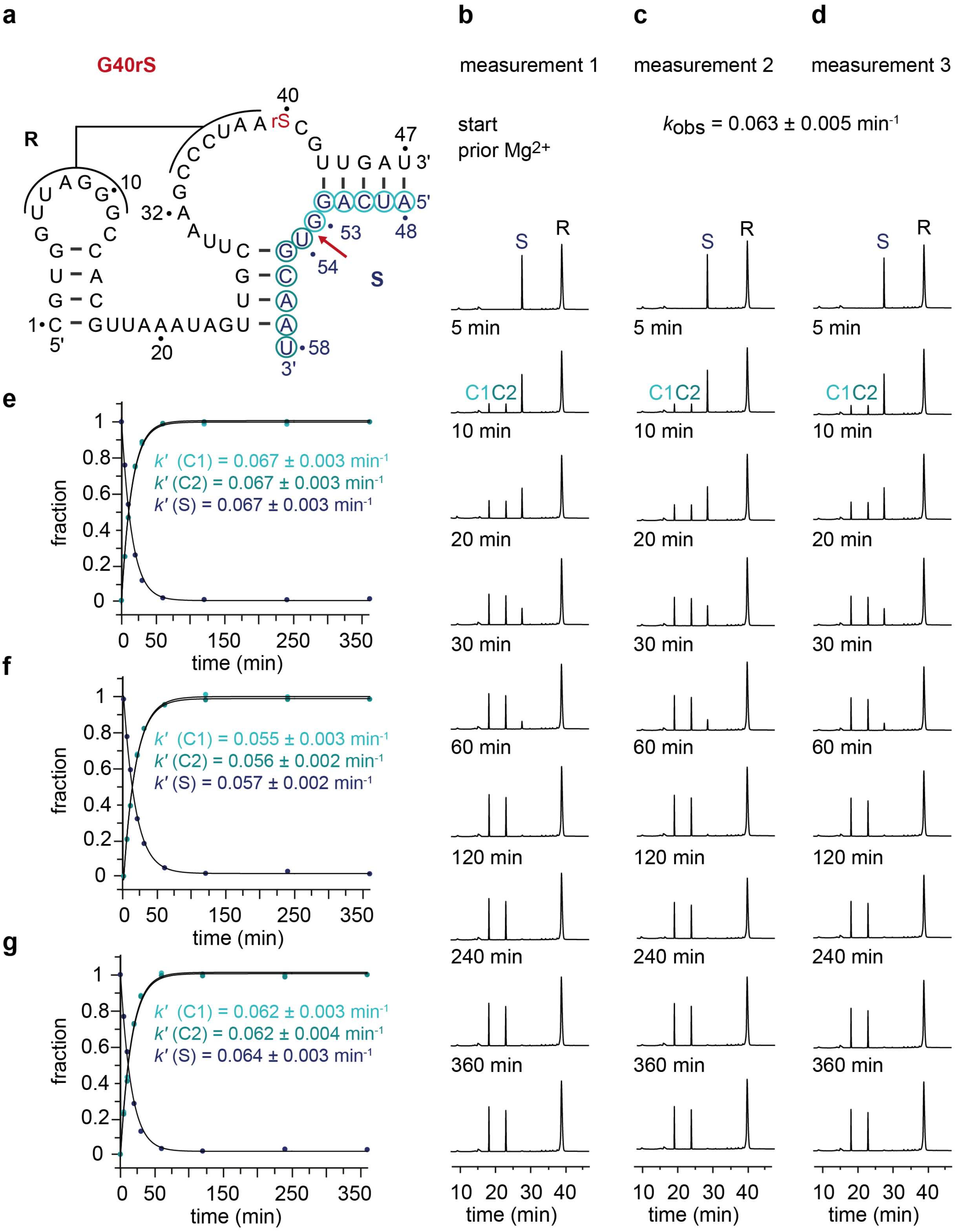

Supporting Figure S15. Self-cleavage of G40rS *env25* pistol ribozyme mutant. **a**) G40rS modified RNA set-up; **b-d**) Anion exchange HPLC profiles of the reaction time course of three independent measurements; reaction conditions: c(RNA) = 55 µM each RNA strand (1:1 ratio); 2 mM $MgCl_2$, 100 mM KCl, 30 mM HEPES, pH 7.5, 25 °C. The reaction was stopped at the indicated time points by drawing a 4 µL sample and mixing it with 4 µL of stop solution (40 mM Na_2EDTA), followed by dilution into 100 µL of water. HPLC conditions: Dionex DNAPac column (4x250 mm), 60 °C, 1 ml min^{-1}, 0–60% buffer B in 45 min. Buffer A: Tris–HCl (25 mM), urea (6 M), pH 8.0. Buffer B: Tris–HCl (25 mM), urea (6 M), $NaClO_4$ (0.5 M), pH 8.0. **d-e**) Estimation of observed rates from fractions (S, C1, and C2) obtained by HPLC analysis.

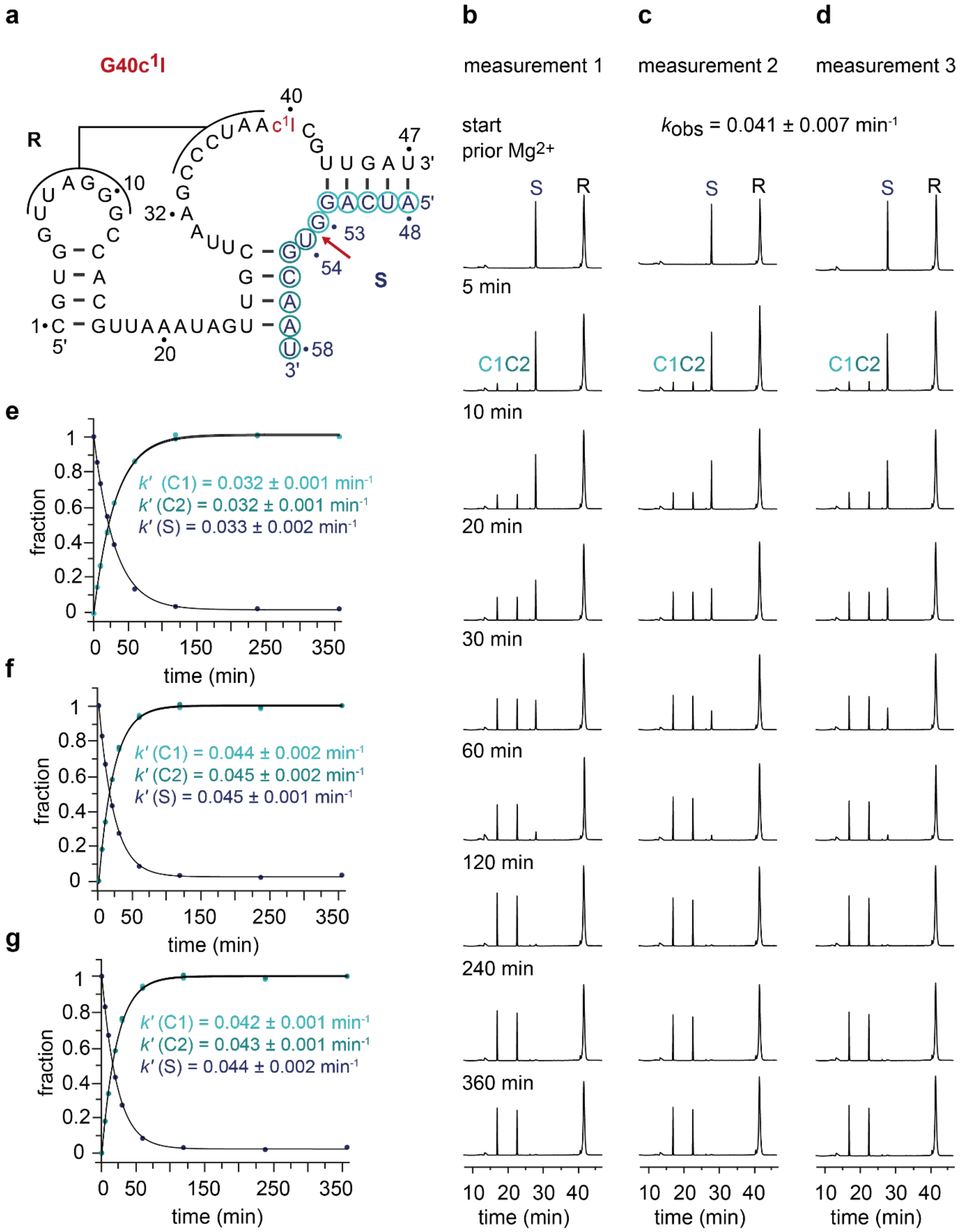

Supporting Figure S16. Self-cleavage of G40c[1]I *env25* pistol ribozyme mutant. **a**) G40c[1]I modified RNA set-up; **b-d**) Anion exchange HPLC profiles of the reaction time course of three independent measurements; reaction conditions: c(RNA) = 55 µM each RNA strand (1:1 ratio); 2 mM $MgCl_2$, 100 mM KCl, 30 mM HEPES, pH 7.5, 25 °C. The reaction was stopped at the indicated time points by drawing a 4 µL sample and mixing it with 4 µL of stop solution (40 mM Na_2EDTA), followed by dilution into 100 µL of water. HPLC conditions: Dionex DNAPac column (4x250 mm), 60 °C, 1 ml min^{-1}, 0–60% buffer B in 45 min. Buffer A: Tris–HCl (25 mM), urea (6 M), pH 8.0. Buffer B: Tris–HCl (25 mM), urea (6 M), $NaClO_4$ (0.5M), pH 8.0. **d-e**) Estimation of observed rates from fractions (S, C1, and C2) obtained by HPLC analysis.

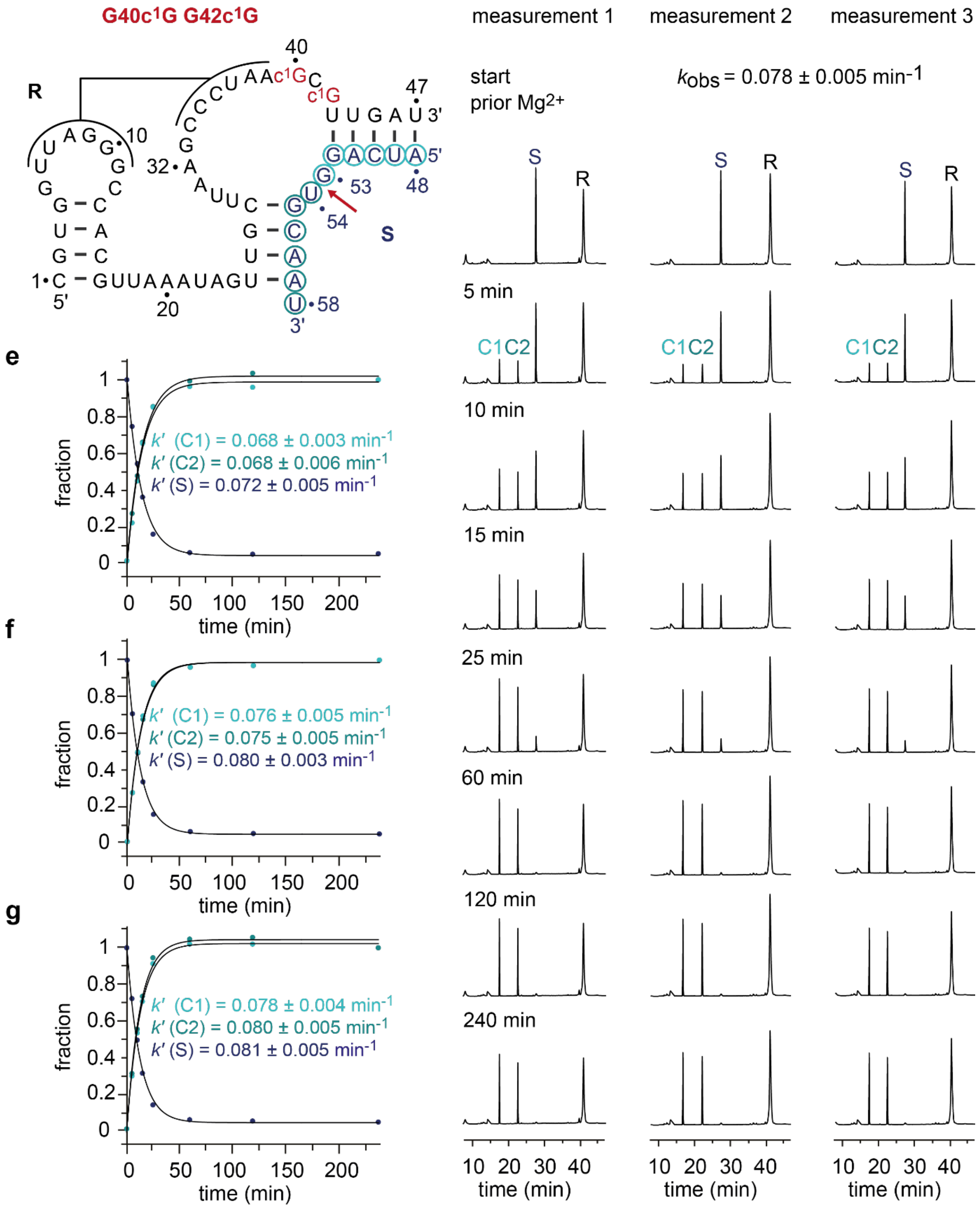

Supporting Figure S17. Self-cleavage of G40c[1]G G42c[1]G *env25* pistol ribozyme mutant. **a**) G40c[1]G G42c[1]G modified RNA set-up; **b-d**) Anion exchange HPLC profiles of the reaction time course of three independent measurements; reaction conditions: c(RNA) = 55 µM each RNA strand (1:1 ratio); 2 mM $MgCl_2$, 100 mM KCl, 30 mM HEPES, pH 7.5, 25 °C. The reaction was stopped at the indicated time points by drawing a 4 µL sample and mixing it with 4 µL of stop solution (40 mM Na_2EDTA), followed by dilution into 100 µL of water. HPLC conditions: Dionex DNAPac column (4x250 mm), 60 °C, 1 ml min^{-1}, 0–60% buffer B in 45 min. Buffer A: Tris–HCl (25 mM), urea (6 M), pH 8.0. Buffer B: Tris–HCl (25 mM), urea (6 M), $NaClO_4$ (0.5 M), pH 8.0. **d-e**) Estimation of observed rates from fractions (S, C1, and C2) obtained by HPLC analysis.

Supporting Table S1. Buffer systems used for cleavage rate-pH profile studies:

FRET assay		HPLC assay	
**pH**	**Buffer**	**pH**	**Buffer**
5 - 6.5	50 mM MES, 100 mM KCl	5 - 6.5	30 mM MES, 100 mM KCl
7 - 7.5	50 mM MOPS, 100 mM KCl	7 - 7.5	30 mM MOPS, 100 mM KCl
8 - 8.5	50 mM TRIS, 100 mM KCl	8 - 8.5	30 mM TRIS, 100 mM KCl
9 - 9.5	50 mM TAPS, 100 mM KCl	9 - 9.5	30 mM TAPS, 100 mM KCl

Abbreviations: MES (2-(*N*-Morpholino)ethansulfonsäure); MOPS (3-(*N*-Morpholino)propanesulfonic acid); TRIS(Tris(hydroxymethyl)aminomethane); TAPS(([tris(hydroxymethyl)methylamino]-propanesulfonic acid).

Supporting Table S2. Components of pistol ribozyme studied in this work.

RNAs	*Sequence* [a]	*Molecular weight* Found[b] *m/z*	Calc *m/z*
	2-Stranded complex used for cleavage assays		
Ribozyme variants			
G40 (wild-type)	5'-CGUGGUUAGGGCCACGUUAAAUAGUU GCUUAAGCCCUAAGCGUUGAU-3'	15081.00	15080.08
G40c3G	5'-CGUGGUUAGGGCCACGUUAAAUAGUU GCUUAAGCCCUAAc3GCGUUGAU-3'	15079.38	15078.93
G40c1G	5'-CGUGGUUAGGGCCACGUUAAAUAGUU GCUUAAGCCCUAAc1GCGUUGAU-3'	15079.80	15078.93
G40X	5'-CGUGGUUAGGGCCACGUUAAAUAGUU GCUUAAGCCCUAAXCGUUGAU-3'	15081.15	15081.06
G40A	5'-CGUGGUUAGGGCCACGUUAAAUAGUU GCUUAAGCCCUAAACGUUGAU-3'	15063.99	15064.08
G40C	5'-CGUGGUUAGGGCCACGUUAAAUAGUU GCUUAAGCCCUAACCGUUGAU-3'	15040.06	15040.05
G40U	5'-CGUGGUUAGGGCCACGUUAAAUAGUU GCUUAAGCCCUAAUCGUUGAU-3'	15040.71	15041.04
G40rS	5'-CGUGGUUAGGGCCACGUUAAAUAGUU GCUUAAGCCCUAArSCGUUGAU-3'	14930.69	14931.05
G40c1I	5'-CGUGGUUAGGGCCACGUUAAAUAGUU GCUUAAGCCCUAAc1ICGUUGAU-3'	15063.69	15064.08
G42c1G	5'-CGUGGUUAGGGCCACGUUAAAUAGUU GCUUAAGCCCUAAGCc1GUUGAU-3'	15078.23	15078.08
G40c1G G42c1G	5'-CGUGGUUAGGGCCACGUUAAAUAGUU GCUUAAGCCCUAAc1GCc1GUUGAU-3'	15077.06	15076.08
Substrate variants			
wild-type	5'-AUCAGGUGCAA-3'	3513.51	3513.21
5'NH_2 3'N_3	5'-NH_2C_6-O-(PO_2)-O-AUCAGGUGCAAU-2'O-N_3-3'	4082.83	4082.63
5'NH_2-G53dG-3'N_3	5'-NH_2C_6-O-(PO_2)-O-AUCAGdGUGCAAU-2'O-N_3-3'	4065.53	4065.91
5'Cy5 3'Cy3	5'-Cy5-NH-C_6-O-(PO_2)-O-AUCAGGUGCAAU-2'O-Cy3-3'	5623.13	5623.54
5'Cy5-G53dG-3'Cy3	5'-Cy5-NH-C_6-O-(PO_2)-O-AUCAGdGUGCAAU-2'O-Cy3-3'	5607.36	5607.36

[a] c3G – 3-deazaguanosine, c1G – 1-deazaguanosine, X – xanthosine, rS – rSpacer, c1I – 1-deazainosine, [b] Reversed-phase LC-ESI mass spectrometry (see Methods).

Supporting Table S3. Pistol ribozyme cleavage kinetics (k_{obs}) of wildtype and mutants.[a]

Pistol variant	k_{obs} (pH 7.5)[b] [min^{-1}]	x-fold relative to wildtype	pK_a (free nucleoside)
G40 (wildtype)	3.79 ± 0.23		9.2 - 9.5 (N1)
c^1G40	1.41 ± 0.08	2.7	9.1 (O6)
c^3G40	0.53 ± 0.12	7.2	12.3 (N1)
X40	0.072 ± 0.003	53	5.7 (N3)
c^1I40	0.041x ± 0.007	92	9.5 (O6)
c^1G42	0.89 ± 0.06	4.2	9.1 (O6)
c^1G42 c^1G40	0.078x ± 0.005	49	9.1 (O6)
A40	0.11x ± 0.02	34	
C40	0.043x ± 0.008	94	
U40	0.025x ± 0.003	152	9.5 -10.5 (N3)
rS40	0.063x ± 0.005	60	
other mutants reported in the literature:			
I40 [c]	1.12 ± 0.02 [c]	8 [c]	8.7 (N1)
I40 [d]	0.018 ± 0.002 [d]	211	8.7 (N1)
2Ap40 [d]	4.21 ± 0.15 [d]		

[a] Cleavage kinetics of wildtype, c^1G, and c^3G pistol were measured using the FRET assay, cleavage kinetics of X40 modified pistol RNA was obtained via HPLC (see Supporting Information). [b] Conditions: 10 mM $MgCl_2$, 100 mM KCl, 50 mM MOPS, pH 7.5, 25 °C. [c] Cleavage rate reported by Wilson, T.J. et al., *J. Am. Chem. Soc.* **2019**, *141*, 7865; note that this rate is 8-fold decreased compared to the rate reported for the wildtype pistol (G40) reported in the same paper (9.8 ± 0.6 min^{-1}). [d] Cleavage rate reported by Teplova, M. et al., *Angew. Chem. Int. Ed.* **2020**, *59*, 2837.

Abbreviations: c^3G – 3-deazaguanosine, c^1G – 1-deazaguanosine, X – xanthosine, c^1I – 1-deazainosine, rS – rSpacer, I – inosine, 2Ap – 2-aminopurine ribonucleoside.

References

1. Santner, T., Hartl, M.., Bister, K., Micura, R. Efficient Access to 3-Terminal Azide-Modified RNA for Inverse Click-Labeling Patterns. *Bioconjugate Chemistry* **25**, 188-195 (2014).

2. Teplova, M., Falschlunger, C., Krasheninina, O., Egger, M., Ren, A., Patel, D. J., Micura, R. Crucial Roles of Two Hydrated Mg^{2+} Ions in Reaction Catalysis of the Pistol Ribozyme. *Angewandte Chemie Soc.* **132**, 2859-28665 (2020).

3. Neuner, S., Falschlunger, C., Fuchs, E., Himmelstoss, M., Ren, A., Patel, D. J., Micura, R. Atom-Specific Mutagenesis Reveals Structural and Catalytic Roles for an Active-Site Adenosine and Hydrated Mg^{2+} in Pistol Ribozymes. *Angewandte Chemie Soc.* **56**, 15954-15958 (2017).

4. Frankel, E. A., Bevilacqua, P. C., Complexity in pH-Dependent Ribozyme Kinetics: Dark pKa Shifts and Wavy Rate-pH Profiles. Biochemistry **57**, 483-488 (2018).

5. Wilson, T. J., Liu, Y., Fuchs, Li, N., Dai, Q., Piccirilli, A., Lilley, D. M., Comparison of the Structures and Mechanisms of the Pistol and Hammerhead Ribozymes. *J. Am. Chem. Soc.* **141**, 7865-7875 (2019).

6. Sam, D., Perona, J. J. Catalytic Roles of Divalent Metal Ions in Phosphoryl Transfer by EcoRV Endocuclease. *Biochemistry* **38**, 6576-6586 (1999).
